# Perceptions of ageism among undergraduate nursing students

**DOI:** 10.1590/1980-220X-REEUSP-2024-0411en

**Published:** 2026-02-23

**Authors:** Vitória da Cruz Moura, Luciana Mitsue Sakano Niwa, Suely Itsuko Ciosak

**Affiliations:** 1 Universidade de São Paulo, Escola de Enfermagem, São Paulo, SP, Brazil.; 2 Universidade de São Paulo, Escola de Enfermagem, Programa de Pós-Graduação em Enfermagem, São Paulo, SP, Brazil.; 3 Universidade de São Paulo, Escola de Enfermagem, Departamento de Enfermagem em Saúde Coletiva, São Paulo, SP, Brazil.

**Keywords:** Ageism, Perception, Nursing, Students, Nursing

## Abstract

**Objective:**

To analyze the social representations of ageism among undergraduate nursing students.

**Method:**

Exploratory and descriptive study, with a quantitative and qualitative approach. Data was collected via Google Forms, investigating the sociodemographic profile of nursing undergraduates in the state of São Paulo and their perceptions of ageism. Quantitative data were subjected to statistical analysis, while the evocations were processed using the software *openEvoc*, based on the Theory of Social Representations (Moscovici, 2003) and analyzed according to Bardin (2015).

**Results:**

A total of 242 students participated, with an average age of 23.8 years; 78.9% (n = 191) were female, 74.0% (n = 179) attended public universities, and 40.1% (n = 97) were in their 3rd year of undergraduate studies. On the whole, 1200 evocations were listed, organized into four categories: “Experience,” “Longevity as a Process,” “Stereotypes,” and “Public Policies.”

**Conclusion:**

Positive perceptions of ageism prevailed, although negative and stereotypical terms were still recurrent. These findings reinforce the need for pedagogical strategies addressing ageism in nursing education, to promote more critical and positive attitudes towards older people.

## INTRODUCTION

Population aging, a phenomenon resulting from the demographic transition characterized by a reduction in mortality and birth rates, has been driven by increased life expectancy coupled with advances in the health and economic sectors^(1)^. This process has resulted in accelerated aging in Brazil, where the proportion of individuals aged 65 or older has increased from 9.84% in 2020 to a projected 31.0% in 2070, tripling in five decades^(2)^. This scenario represents a social achievement, but it poses significant challenges, especially to health systems, demanding the formulation of integrated public policies that promote healthy aging. Furthermore, the construction of favorable social and cultural environments capable of combating stereotypes that associate aging with illness, dependency, and uselessness is required^(3,4)^.

This age-related prejudice is called *idadismo, ageísmo* or *etarismo* in Portuguese, and in English ageism, which is defined as:

“…stereotypes (the way we think), prejudices (how we feel), and discrimination (how we act), based on age, directed at others or against oneself. Ageism can manifest itself at the institutional or interpersonal level and can be self-directed (against oneself). Ageism can be implicit or explicit, depending on the level of awareness of our own bias”^(4)^.

According to the Pan American Health Organization (PAHO), ageism against older people is a global phenomenon, manifesting itself in all institutions of society such as health, social assistance, work, and the media, where they are often underrepresented. This practice compromises well-being, hinders the implementation of effective policies and actions aimed at promoting the health of the older population, and represents a challenge for care, which should prioritize quality of life, even in the face of age-related limitations. This goal becomes achievable as society recognizes the value and potential of older people, overcoming barriers resulting from social devaluation^(3,4)^.

In response, PAHO published the World Report on Ageism, outlining evidence-based strategies to reduce the phenomenon and its impacts^(4)^. However, stereotypes and prejudices directed at older people, frequently reproduced in the daily lives of healthcare professionals, negatively influence care and problem-solving, increasing the vulnerability of this population^(5)^.

Ageism in healthcare manifests itself, among other aspects, in the neglect of the sexuality of older adults, a topic which many professionals do not feel prepared to address^(5,6)^. There is also evidence of age-based rationing of care, as demonstrated by a systematic review that identified age as a determining factor in access to specific medical procedures or treatments in 85% of 149 studies^(7)^. Adding to this is the scarcity of health studies including the older population, despite the high prevalence of the conditions studied in these individuals^(4)^. The impacts of ageism are associated with reduced life expectancy, worsening physical and mental health, slow recovery from disabilities, cognitive decline, and reduced quality of life, with increased isolation and loneliness.

Studies on teaching and learning show gaps in nursing education regarding the health of older adults, including deficits in the lack of uniformity in research on ageism throughout undergraduate studies: a 2023 systematic review identified inconsistent results on ageism during undergraduate studies, with nine studies indicating greater positivity throughout the course and seven studies showing no significant differences, finding failures in the positive progression of perceptions over the years of undergraduate students at the educational institution^(8)^.

Despite the recognition of the topic relevance, scientific production is predominantly quantitative, as shown by a scoping review of 46 studies, of which 73.9% were quantitative, 10.8% qualitative, and 6.6% mixed^(9)^. In this sense, qualitative analysis in the field of older people health is of great value, especially in understanding the meanings, experiences, and behaviors— aspects that are difficult to access through purely quantitative analysis^(10)^. Furthermore, national studies indicate that the inclusion of older people health in Nursing education remains incipient, restricted to isolated initiatives and poorly consolidated strategies^(11)^. This highlights the need to broaden the scientific and pedagogical debate on ageism in undergraduate education, supporting mixed analyses and deepening discussions on the subject.

It should be noted that the theoretical framework used was Serge Moscovici’s theory of social representations, where it is essential to discuss the concepts of anchoring and objectification, processes that generate social representations. Moscovici proposes that anchoring consists of transforming the unfamiliar into something familiar, through the classification and categorization of the unknown based on what is already known. Objectification, in turn, would be transforming the abstract into something almost concrete^(12)^.

To comprehend how society is understood, Moscovici divides the universe into consensual and reified. The consensual realm is where social representations are formed, encompassing common sense. The reified, in turn, corresponds to the field of scientific knowledge^(12)^. In this regard, perception leans towards one of these universes, varying according to the social group to which one belongs or in which one is embedded.

The theory of social representations is prominently featured in the field of health. Those who provide care are bearers of scientific knowledge, including nurses; however, it must be considered that these bearers of scientific knowledge belong to a social group and are subject daily to influences from the different environments of the society to which they belong.

Thus, the present study aims to provide evidence that contributes to reducing the identified gap and had the objective of knowing and analyzing the social representations of undergraduate nursing students regarding ageism and characterizing the profile of the students in the sample, since they, as future nurses, will work in the care of older people and must understand and demystify ageism, with undergraduate studies playing a vital role in this context.

## METHOD

### Design of Study

To better address the objectives, aiming to understand the perceptions of Nursing students regarding ageism, the present study had an exploratory and descriptive character, with quantitative and qualitative data methodology. The Content Analysis technique, based on Serge Moscovici’s Theory of Social Representations, was used as a theoretical and methodological framework^(13)^.

### Local

The research was conducted in a virtual environment, via an online Google Forms questionnaire.

### Population and Selection Criteria

Participants were undergraduate nursing students who entered the program from 2021 onwards, self-identified as being of both sexes, over 18 years of age, and enrolled in public and private undergraduate institutions in the state of São Paulo. Former students and underage graduates were excluded.

### Data Collection Instrument

A Google Forms questionnaire was created with questions about the student’s profile and open-ended questions about their perceptions of ageism.

### Data Collection Procedure

Data collection was carried out from March to June 2024, using the “Snowball” chain sampling technique, in which participants were recruited from the researchers’ contacts and subsequently asked to indicate new contacts with the desired characteristics. The form was sent via email and/or WhatsApp, and answered anonymously after virtual acceptance of the Free and Informed Consent Form (FICF), indexed at the beginning of the form. The average time to complete the instrument was 7 minutes, and the questionnaire was answered in a single round, without subsequent feedback for validation or supplementation of the qualitative data.

### Data Analysis and Treatment

The quantitative data were recorded in Excel^®^ spreadsheets and presented in frequency tables. The evocations were processed using OpenEvoc software for qualitative analysis and then examined using Bardin’s content analysis to interpret the meanings attributed by the participants based on the Theory of Social Representations^(14)^.

### Ethical Aspects

In accordance with the National Health Council Resolution No. 466/2012, this project was approved by the Research Ethics Committee of the USP Nursing School, Opinion No. 6.691.256, and, in accordance with the same Resolution, all participants read and signed the FICF^(15)^. To ensure privacy, all participants were identified with the letter G followed by sequential numbers (G1, G2, G3…).

## RESULTS

### Sociodemographic Profile of Nursing Students

The study included 308 undergraduate nursing students, of whom 242 met the inclusion criteria and comprised the final sample. The undergraduates were between 18 and 51 years old (mean 23.8 and median 21.5), predominantly female (78.9%; n = 191), from public universities (74.0%; n = 179) and in their 3rd year of undergraduate studies (40.1%; n = 97). In this group, 5.4% (n = 13) of the participants completed another degree before their nursing degree, and 24.4% (n = 59) were working concurrently with their degree. It is worth highlighting that of these, 65.1% (n = 41) are from private universities. Most respondents, 80.6% (n = 195), interacted with older people on a daily basis, and 80.2% (n = 194) were familiar with the concept of ageism (Table 1).

**Table 1:** Profile data of nursing undergraduates – São Paulo, SP, Brazil, 2024.

Variables	Public University		Private University		Total number of participants	
	n	%	n	%	n	%
**Sex**						
Female	138	77.1	53	84.1	191	78.9
Male	39	21.8	10	15.9	49	20.3
Prefers not to identify	2	1.1	0	0.0	2	0.8
**Year of Graduation**						
1^st^ year	50	28.0	13	20.7	63	26.0
2^nd^ year	31	17.3	9	14.3	40	16.5
3^rd^ year	77	43.0	20	31.7	97	40.1
4^th^ year	21	11.7	21	33.3	42	17.4
**Another Degree**						
Yes	6	3.4	7	11.1	13	5.4
No	173	96.6	56	88.9	229	94.6
Work						
Yes	18	10.1	41	65.1	59	24.4
No	161	89.9	22	34.9	183	75.6
**Have a close relationship with older people**						
Yes	140	78.2	55	87.3	195	80.6
No	39	21.8	8	12.7	47	19.4
**Knows the meaning of ageism**						
Yes	153	85.5	41	65.1	194	80.2
No	26	14.5	22	34.9	48	19.8
Total	179	100.0	63	100.0	242	100.0

The sociodemographic data of the participants were presented in frequency tables with an analytical comparison between public and private units, when significant data were evaluated in the comparison of the sociodemographic profile within the institutions.

Furthermore, the majority, 61.2% (n = 148), of nursing students consider themselves “realistic with some biases”; followed by 25.2% (n = 61) “optimistic and free of biases”; 8.7% (n = 21) “without biases”; and 2.9% (n = 7) “pessimistic and biased”. It should be noted that, among graduates from public universities, the majority positioned themselves as “Realistic, with some biases” (69.3%; n = 124). Graduates from private universities were mostly divided between “Optimistic and free of prejudices”, with 39.7% (n = 25), and “Realistic, with some prejudices”, with 38.1% (n = 24) (Table 2).

**Table 2:** Perceptions from nursing graduates about older people – São Paulo, SP, Brazil, 2024.

Variables	Public University		Private University		Total number of participants	
	n	%	n	%	n	%
Realistic, with some prejudice	124	69.3	24	38.1	148	61.2
Optimistic and free of prejudice.	36	20.1	25	39.7	61	25.2
Without prejudice	9	5.0	12	19.0	21	8.7
Pessimistic and prejudiced	6	3.4	1	1.6	7	2.9
Others	4	2.2	1	1.6	5	2.0
Total	179	100.0	63	100.0	242	100.0

### Expressions about Ageism among Nursing Students

In an effort to identify the social representation of ageism, undergraduate students were asked to name five words/expressions they associated with older people. Some participants submitted more than five words, while others submitted fewer, resulting in 1,200 citations, which were entered into the openEvoc 1.0 software.

Although there were significant differences between private and public institutions in the sociodemographic profile and self-reported perceptions of the undergraduates, there were no relevant differences in their evocations. In the evocations, 58.7% (n = 37) of the undergraduates from private institutions presented perceptions considered positive about the older people and the aging process, while in public institutions it was 63.6% (n = 114). By applying the chi-square test, with a p-value equal to 0.53 > 0.05, there was no statistically significant difference between the types of institutions regarding the connotation of the responses.

Of the total representations, the words and their derivations that stood out the most were: experience (112), wisdom (66), care (41), retirement (32), knowledge (31), fragility (29), life experience (24), respect (22), loneliness (22), and family (20). When asked which of the five words mentioned were most representative, the ones that stood out were: experience, wisdom, care, fragility, and neediness.

Based on the reading and frequency of words and expressions described by the interviewees, it was possible to classify them into 4 categories: Experience, Longevity as a Process, Stereotypes and Public Policies, whose subcategories and units of registration are presented in Chart 1:

**Chart 1:** Expressions from nursing graduates about older people – São Paulo, SP, Brazil, 2024.

Category	Subcategory	Registration unit
1. Experience 25.1% (n = 301)	a. Life experiences (247)	– Experience – Wisdom
	b. Sharing experiences (54)	– Stories – Conversation
2. Longevity as a process 30.7% (n = 368)	a. Vulnerability (227)	– Weaknesses – Illnesses
	a. Physical characteristics (100)	– Aging – Gray hair
	b. Stability (41)	– Rest – Routine
3. Stereotypes 27.6% (n = 332)	a. Personality (192)	– Stubbornness – Patience – Neediness
	b. Childish look (13)	– Cute – Old man
	c. Evocation of the past (44)	– Bingo – “In my day...”
	d. Family ties (83)	– Family – Kindness
4. Public policies 16.6% (n = 199)	a. Promotion (176)	– Care – Retirement
	b. Omission (26)	– Abandonment – Prejudice

1. **Experience:** Gathered the evocations related to wisdom and knowledge acquired by older people through their “Life Experiences” and the way they “Share Experiences” through stories and conversations, propagating their legacy and traditions, with a total of 25.1% (n = 301) of evocations. It can be inferred that this topic is relevant because it includes the two most frequently used words among the evocations: “experience” (112) and “wisdom” (66). When asked about the reason for choosing the words evoked as most representative, the perceptions were presented according to the accounts:

*(**Experience)** is a particularly representative word when referring to an older person because it reflects the accumulation of experiences, learning, and knowledge throughout a lifetime. An older person’s life experience is unique and can offer valuable perspectives on various aspects, such as family, work, society, and overcoming challenges. Furthermore, the experience can be shared to benefit younger generations, contributing to the preservation of traditions, values, and knowledge*. – (G103)

*(**History)** Because it encompasses the burden of life, the way of communicating, the accumulated experience, the passing down of traditions, coexistence and its marks, all in a single word. Even though it’s short, it captures the beauty and pain that old age represents in my view*. – (G162)

2. **Longevity as a process:** This category presents the repercussions related to the period of life in which the older person lived and lives, with the highest percentage, of 30.7% (n = 368) of evocations. The category was subdivided into 3 subcategories: “Vulnerability,” “Physical characteristics”, and “Stability.”

“Vulnerability” was the most frequently cited subcategory, at 61.7% (n = 227), associating the older person with negative words, referring to disabilities and limitations that can make the older person dependent and reduce their quality of life. The subcategory was marked by illnesses, pain, physical limitations, but also by psychological factors such as loneliness, related to depression and anxiety, as in the following statements:

*(**Fragility)** In my opinion, older people are more fragile in their lives, both in terms of physical and psychological health, and in other daily needs*. – (G42)

*(**Loneliness)** I believe that most older people often feel that their families put them second, and this leads them to live in a world where their only possible company is themselves*. – (G57)

The subcategory “Physical characteristics” was related to the appearance and conditions of older people, such as gray hair and the emergence of wrinkles. Furthermore, there are also the terms used to refer to older people based on their characteristics, such as being called “old man/woman” and “sir/madam,” as follows:

*(**Aging)** I chose this word because the results of the aging process are more apparent in older people, such as gray hair and more sensitive skin; in other words, it is possible to observe the marks of the passage of time in them, which is quite interesting because it shows how, in a way, change is part of life*. – (G56)

The subcategory “Stability” represents an idealized view of aging, associating old age with tranquility, rest, and freedom after many years of work. Moreover, the view that disrupting this calm environment could lead to discontent among older people, as can be seen in:

*(**Routine)** Older people generally dislike commitments that disrupt their routine*. – (G166)

*(**Tranquility)** Age brings tranquility through the reduction of daily responsibilities and obligations*. – (G151)

3. **Stereotypes:** This category represented 27.6% (n = 332) of words/expressions and grouped together evocations that represented different stereotypes of older people. “Personality,” “Childlike gaze,” “Evocation of the past,” and “Family ties.” Personality traits emerged in positive ways, such as patience and kindness, but also in negative ways, such as stubbornness and neediness.

*(**Kindness)** Because older people, even strangers, tend to be kind and welcoming*. – (G218)

*(**Stubborn)** Older adults often don’t accept that they are aging, so they don’t accept help and can’t easily perceive their new limitations*. – (G126)

*(**Infantilization)** People infantilize older people*. – (G186)

The “childish gaze” of undergraduate students towards older people, reinforced by diminutive terms (in Portuguese: *velhinho, vôzinho*) used to infantilize the elderly, such as “old man” and “grandpa” in English. The “Evocation of the Past,” with words that refer to old age—bingo, church, lunches at grandparents’ houses, and even habits like recalling the past to compare with the present—can be observed in the following quotations:

*(**Church)** The older population in Brazil is predominantly made up of people with Christian ideology. This ideology is very evident in the way they view the new generation and society as a whole*. – (G218)

*(**In my day…)** Because it denotes birth in another era, different knowledge, ways of living and dealing with the world, it denotes the passage of time*. – (G158)

Also, “Family Ties” describes the relationship of the undergraduate students with older people in their family, such as grandparents, etc., as can be seen in the statement of one student:

*(**Affection)** The word “affection” represents the fondness I have for the older people in my life, and for those in general. If we think about it, thanks to them we are here today; the entire culture of a society, the formation and unity of families, resulted from the choices of our grandparents, and before them, our great-grandparents, and so on. That’s why I believe the word affection can represent them*. – (G55)

4. **Public Policies:** Presented a total of 16.6% (n = 199) of the words/expressions, where two subcategories were distinguished, “Promotion” and “Omission”. In the “Promotion” subcategory, 88.4% (n = 176) of the words mentioned public policies aimed at older people, especially in health and other social areas:

*(**Care)** For me, care encompasses several aspects, such as special attention to chronic non-communicable diseases, which become more common in old age, and the increased risk of falls. The loneliness and abandonment that many older people face also requires greater attention and changes from society*. – (G2)

*(**Respect)** Because the person has more life experiences to share, because they’ve been through so much, because sometimes they aren’t heard or are neglected*. – (G119)

In the “Omission” subcategory, participants associated the older person with vulnerability caused by a lack of public policies, using negative words to denounce adverse situations faced by older people:

(**Social Vulnerability)** I believe that in the society we live, social *vulnerability is inherent to the older person, since their rights are violated daily, whether due to insufficient pensions, lack of accessibility in public spaces, among other reasons*. – (G65)

*(**Invisibility)** I believe that older people are often ignored and made invisible by society. For example, when we consider the poor urban infrastructure that hinders or even prevents the movement of elderly people. Furthermore, there is a lack of public policies aimed at this age group*. – (G78)

## DISCUSSION

The group of undergraduate students who collaborated on the research consisted of a larger number of self-identified females, both in research focused on nursing students within the context of ageism^(9)^ or outside of it. This demonstrates a feminization of nursing, stemming from the category’s history, in which caregiving was seen as a uniquely feminine skill.

It was also found that most participants from private institutions worked concurrently with their undergraduate studies, while those from public institutions were a minority, which is consistent with data from the Union of Higher Education Institutions - SEMESP - regarding the profile of higher education students in Brazil^(16)^. These high figures, focused on undergraduates from private institutions, relate to the need to work to pay for college, occasionally made possible by the institution’s shorter class hours, which allows for evening classes, providing access to work, or even an incentive from the employer for the student to pursue better positions in their career plan.

It is noteworthy that sex did not have a significant influence on the prevalence of the words evoked, a result similar to that found in a cross-sectional study conducted with nursing undergraduates on ageism in Jordan^(15)^. Conversely, other investigations have identified sex-related differences, showing a greater predominance either among female participants or among male participants^(8)^.

The type of educational institution or the year of their undergraduate studies did not affect the prevalence of word placement. Similarly, living with older people and knowing the meaning of ageism also did not show differences in the citations, demonstrating, in a way, common sense regardless of sex, type of institution and year of graduation, unlike the results presented by Allué-Sierra et al.^(8)^, where regular contact or cohabitation with an older person was among three of the main determinants in the expression of positive attitudes towards older people.

Older people’s life experience has been identified as a relevant concept in the social representations manifested, ranging from the acquisition of wisdom from life experiences to the transmission of learning and traditions acquired over time. This initial evocation of the older person as someone experienced has also been identified in other qualitative research on ageism and with undergraduate nursing students, in studies of national (Ceará) and international (Korea) scope^(17,18)^.

Another study analyzed older adults’ representations of their experiences and concluded that it is important for nurses to consider the relevance of older adults’ life experiences for providing adequate and humane patient care, not emphasizing purely procedural care^(19)^.

Therefore, considering the experiences of older people is important, but unfortunately, there is a myth that long-lived people are wise. This representation perpetuates a belief that experience accumulated over the years brings a deeper understanding of life. Although many older people have vast knowledge, wisdom is not exclusive to old age, nor is it mandatory with such advancement^(20)^. In old age, wisdom can manifest itself in various ways, such as the ability to reflect on the past with discernment and the skill to deal with challenges in a more thoughtful manner. Furthermore, it is possible that the undergraduates, in seeking to avoid an ageist perspective, may have placed excessive value on the wisdom of older people, reflecting an attempt to distance themselves from consensual stereotypes associated with aging, thus characterizing a possible bias.

Additionally, society views the aging process as linked to loneliness and dependency; however, this preconception is also often reinforced by healthcare services themselves^(21)^. The research represented longevity as a process, and within that process, the view of the older person as interconnected with vulnerability and frailty stood out. The view of vulnerability in older people is common sense in this and other research, such as in a study conducted with a self-reported questionnaire among undergraduate nursing students^(22)^. However, it is important to remember that many people reach old age with a good quality of life, independence, and autonomy. Therefore, the concept of healthy aging, in which the older person is the protagonist of their actions and choices, still needs to be instilled in undergraduate students, as well as the role of the nurse in prevention and health promotion instead of only treating diseases, disabilities, and limitations.

In this sense, stereotypical perceptions about older people are everywhere, whether in the media, in preferential treatment, or in the health sector. In this study, several stereotypes were associated with older people, such as positive personality traits (patience, cheerfulness, and kindness) and negative traits (stub-bornness, neediness, and conservatism). A childish gaze of the older people was also mentioned, as well as activities related to old age such as bingo, reminiscing about the past, and family ties. In light of this, it should be highlighted that, although negative stereotypes were identified, positive ones were the most frequent, as pointed out in a scoping review. Among the 46 articles analyzed, 23.5% presented positive attitudes and perceptions, representing the largest proportion of studies, followed by negative (19.6%), mixed (19.6%), neutral (4.3%), and inconclusive (6.5%) studies^(9)^.

Stereotypes about old age are still prevalent and have harmful consequences for society, as they compromise self-esteem and, consequently, individuals’ physical and mental health. Furthermore, they result in the exclusion of people from social and work spaces, generating negative impacts in various sectors of life^(23)^. Although the study in question and other studies show that undergraduates have greater contact with older family members^(24,25)^, it is necessary to broaden this perspective to provide adequate care, free from prejudice, and that promotes healthy aging.

Furthermore, regarding physical characteristics, wrinkles, gray hair, and sagging skin mark the phenotype of older people, as presented in the representations of this and other studies^(22)^. However, in Brazil, there is a certain movement towards aging without looking like an older person, since, according to Castro et al.^(26)^, wrinkles and gray hair, characteristics mentioned in the studies, are considered a sign of neglect regarding one’s appearance. Thus, the common physical characteristics resulting from the aging process manifest themselves negatively by interfering with the self-esteem of the person who is transitioning from adulthood to old age.

The students evocation that stood out was that about the importance of public policies focused on health and life in society, which are often neglected but fundamental to promoting healthy aging. In another study of social representations, this dimension related to public policies emerged after the application of classes on older health, not having been initially identified among the participants^(17)^.

Negative aspects were reported, such as the denial of rights, which increase older people’s vulnerability. The WHO defines healthy aging as the process of developing and maintaining functional capacity that allows for well-being in old age, emphasizing the importance of policies that promote social inclusion, accessibility to health services, and support for lifestyles that favor the autonomy and independence of older adults^(27)^.

These aspects are reinforced by the Statute of the Older Person, in which, according to Article 1, “The Statute of the Older Person is hereby established, intended to regulate the rights guaranteed to persons aged 60 (sixty) years or older,” supporting the participants’ representations regarding public policies aimed at older people^(28)^.

The importance of these evocations is considered, since, as future nurses, they should also be political agents in the health field, acting in defense of the older people and in empowering their peers to make changes, considering the older people with their vulnerabilities, potentialities and needs, to improve their quality of life, at all levels of care in the territory where they will practice their role.

The study’s strengths lie in the originality and relevance of the topic, which is still relatively unexplored in nursing education, coupled with a consistent methodology that combines quantitative and qualitative approaches grounded in the Theory of Social Representations. It has a significant sample size, which lends robustness to the findings, organized into broad categories that reveal both positive and negative perceptions about ageism. Moreover, the inclusion of the public policy dimension stands out as an innovative aspect in the field, along with practical contributions to pedagogical strategies, reinforcing the importance of critical and inclusive training for future nurses. Despite its contributions, this study has limitations that should be considered, such as the use of a convenience sample, restricted to undergraduate nursing students, which limits the extrapolation of the results. Likewise, the online data collection may have favored social desirability bias and, because it is a cross-sectional design, it does not allow establishing causality, only associations between the variables analyzed. Future research, with larger samples and in diverse contexts, could deepen and broaden the understanding of ageism in the context of health education.

## CONCLUSION

According to the analysis, it was possible to identify that most nursing students are aware of the concept of ageism and appear to be open to learning more about the topic, presenting optimistic or realistic perceptions about it. Although the word “ageism” is not directly mentioned in the perceptions, they evoke current societal issues that permeate the older person and the aging process in various areas, such as public policies and healthcare. In addition, these perceptions also associate the older person with feelings considered positive, such as family ties and qualities like wisdom, experience, and patience.

However, several negative words still appear in the participants’ minds about older people, and various stereotypes are evoked, such as negative physical and personality characteristics, as well as the association of older people with illness, vulnerability, and an infantilized view.

Therefore, it is extremely important that knowledge about ageism be addressed and reinforced among undergraduate students, especially nursing students, who may be in direct and continuous contact with older people, to maintain a positive feeling towards them, since they are a growing population worldwide and will certainly be the target of care, whether in the preventive or assistance area, at the outpatient, institutional or home level. Furthermore, the goal is for students and future nurses to become representatives and multipliers in the fight against ageism in society and in their future professional lives.

## Data Availability

The entire dataset supporting the results of this study was published in the article itself.

## References

[1] Miranda GMD, Mendes ACG, Silva ALA (2016). Population aging in Brazil: current and future social challenges and consequences. Rev Bras Geriatr Gerontol.

[2] Instituto Brasileiro de Geografia e Estatística (2025). Projeções da população do Brasil e Unidades da Federação: 2000-2070: população e sexo por idade. Rio de Janeiro: IBGE.

[3] Ciosak SI, Braz E, Costa MFBNA, Nakano NGR, Rodrigues J, Alencar RA (2011). Senescence and senility: a new paradigm in Primary Health Care. Rev Esc Enferm USP.

[4] Organização Pan-Americana da Saúde (2022). Relatório mundial sobre o idadismo.

[5] Silva MF, Silva DSM, Bacurau AGM, Francisco PMSB, Assumpção D, Neri AL (2021). Ageism against older adults in the context of the COVID-19 pandemic: an integrative review. Rev Saude Publica.

[6] Alencar RA, Ciosak SI (2016). Aids in the elderly: reasons that lead to late diagnosis. Rev Bras Enferm.

[7] Chang ES, Kannoth S, Levy S, Wang SY, Lee JE, Levy BR (2020). Global reach of ageism on older persons’ health: a systematic review. PLoS One.

[8] Allué-Sierra L, Antón-Solanas I, Rodríguez-Roca B, Anguas-Gracia A, Echániz-Serrano E, Fernández-Rodrigo MT (2023). Ageism and nursing students, past or reality? A systematic review. Nurse Educ Today.

[9] Fhon JRS, Alves N, Santos No AP, Djinan ARFS, Laurenti AV (2024). Attitudes and perceptions of ageism among nursing students: a scoping review. Rev Lat Am Enfermagem.

[10] Cyriaco AFF, Nunn D, Amorim RFB, Falcão DP, Moreno H (2017). Pesquisa qualitativa: conceitos-chave e uma breve visão geral de sua aplicação em geriatria/gerontologia. Geriatr Gerontol Aging.

[11] Oliveira ACLC (2023). A formação dos enfermeiros para a atenção á saúde da população idosa [dissertação]. Natal: Universidade Federal do Rio Grande do Norte.

[12] Cândido CM, Assis MR, Ferreira NT, Souza MA (2014). A representação social do “bom professor” no ensino superior. Psicol Soc.

[13] Moscovici S (2011). Representações sociais: investigações em psicologia social. Petrópolis: Vozes.

[14] Bardin L (2011). Análise de conteúdo. São Paulo: Edições 70.

[15] Rababa M, Al-Dwaikat T, Almomani MH (2021). Assessing knowledge and ageist attitudes and behaviors toward older adults among undergraduate nursing students. Gerontol Geriatr Educ.

[16] Instituto SEMESP (2025). Mapa do Ensino Superior no Brasil. São Paulo.

[17] Alexandre SG, Silva HG, Silva VMGN, Rocha VA, Lopes JP, Freitas MC (2016). Conceptions of nursing undergraduate students about the elderly and old age. J Nurs UFPE on line.

[18] Jo K-H, An G-J (2012). Perception of aging among Korean undergraduate nursing students. Acta Paul Enferm.

[19] Queiroz LMP, Lazarini CA, Higa EFR, Pinto AAM (2024). Social representations of Family Health Strategy professionals about palliative care for older adults. Rev Bras Geriatr Gerontol.

[20] Grossmann I, Gerlach TM, Denissen JJA (2016). Wise reasoning in the face of everyday life challenges. Soc Psychol Personal Sci.

[21] Quaresma ML, Ribeirinho C (2016). Envelhecimento: desafios do séc. XXI. Rev Kairós.

[22] Moyle W (2003). Nursing students perceptions of older people: continuins society’s myths. Aust J Adv Nurs.

[23] Machado MH (2017). Relatório final da pesquisa perfil da enfermagem no Brasil. Rio de Janeiro: FIOCRUZ/COFEN.

[24] Attafuah PYA, Amertil N, Sarfo JO, Deegbe DA, Nyonator D, Amponsah-Boama C (2022). ‘Decidi atendê-lo porque é meu dever’: percepção e atitude dos estudantes de enfermagem em relação ao cuidado de idosos. BMC Med Educ.

[25] Fu Y, Zhang J, Cao L, Ma J, Zhu H, Dong Y (2022). Nursing students’ attitudes and associated factors towards older people in Heilongjiang Province, northern China: a cross-sectional study. Nurs Open.

[26] Castro A, Antunes L, Maldonado Brito AM, Camargo BV (2016). Representações sociais do envelhecimento e do rejuvenescimento para mulheres que adotam práticas de rejuvenescimento. PSICO.

[27] Organização Mundial da Saúde (2015). Relatório mundial de envelhecimento e saúde. Brasília: OMS.

[28] Brasil. Lei n 14.423, de 22 de Julho de 2022 (2022). Dispõe sobre o Estatuto da Pessoa Idosa e dá outras providências. Altera a Lei nº 10.741, de 1º de outubro de 2003, para substituir, em toda a Lei, as expressões “idoso” e “idosos” pelas expressões “pessoa idosa” e “pessoas idosas”, respectivamente. Diário Oficial da União; Brasília.

