# Supplementary material for: Perceptions of ageism among undergraduate nursing students
Source: Rev Esc Enferm USP. 2026 Feb 23;60:e20240411. doi: 10.1590/1980-220X-REEUSP-2024-0411en (PMC12945305; doi:10.1590/1980-220X-REEUSP-2024-0411en)
Supplement: Translation

# Translation: Percepções sobre idadismo em estudantes de graduação em enfermagem

**DOI:** 10.1590/1980-220X-REEUSP-2024-0411pt

**Authors:** Vitória da Cruz Moura, Luciana Mitsue Sakano Niwa, Suely Itsuko Ciosak

**Affiliations:** 1 Universidade de São Paulo, Escola de Enfermagem, São Paulo, SP, Brasil.; 2 Universidade de São Paulo, Escola de Enfermagem, Programa de Pós-Graduação em Enfermagem, São Paulo, SP, Brasil.; 3 Universidade de São Paulo, Escola de Enfermagem, Departamento de Enfermagem em Saúde Coletiva, São Paulo, SP, Brasil.

**Keywords:** Etarismo, Percepção, Enfermagem, Estudantes de Enfermagem

## Abstract

**Objetivo:**

Analisar as representações sociais de estudantes de graduação em Enfermagem sobre o idadismo.

**Método:**

Estudo exploratório e descritivo, com abordagem quantitativa e qualitativa. Os dados foram coletados via Google Forms, investigando o perfil sociodemográfico dos graduandos de Enfermagem do estado de São Paulo e suas percepções sobre o idadismo. Dados quantitativos foram submetidos à análise estatística, enquanto as evocações foram processadas no software *openEvoc*, com base na Teoria das Representações Sociais (Moscovici, 2003) e analisados segundo Bardin (2015).

**Resultados:**

Participaram 242 estudantes, média de idade de 23,8 anos; 78,9% (n = 191) do sexo feminino, 74,0% (n = 179) de universidades públicas e 40,1% (n = 97) do 3º ano da graduação. Foram elencadas 1200 evocações, organizadas em quatro categorias: “Vivência”, “Longevidade como processo”, “Estereótipos” e “Políticas públicas”.

**Conclusão:**

Predominaram percepções positivas sobre o idadismo, embora termos negativos e estereotipados ainda tenham sido recorrentes. Tais achados reforçam a necessidade de estratégias pedagógicas que abordem o idadismo na formação em Enfermagem, de modo a promover atitudes mais críticas e positivas em relação às pessoas idosas.

## INTRODUÇÃO

O envelhecimento populacional, fenômeno decorrente da transição demográfica caracterizada pela redução das taxas de mortalidade e natalidade, tem sido impulsionado pelo aumento da expectativa de vida associado a avanços nos setores de saúde e economia^(1)^. Esse processo resultou em um envelhecimento acelerado no Brasil, cuja proporção de indivíduos com 65 anos ou mais passou de 9,84% em 2020 para uma projeção de 31,0% em 2070, triplicando em 5 décadas^(2)^. Tal cenário representa uma conquista social, porém impõe desafios significativos, sobretudo aos sistemas de saúde, demandando a formulação de políticas públicas integradas que promovam o envelhecimento saudável. Além disso, faz-se necessária a construção de ambientes sociais e culturais favoráveis, capazes de combater estereótipos que associam o envelhecimento à doença, dependência e inutilidade^(3,4)^.

Esse preconceito relacionado à idade é denominado idadismo, ageísmo ou etarismo e nesta pesquisa, iremos adotar idadismo, cuja definição:

“… se refere a estereótipos (a maneira como pensamos), preconceitos (como sentimos) e a discriminação (como agimos), com base na idade, dirigidos a outros ou contra si mesmo. O idadismo pode se manifestar no nível institucional ou interpessoal e pode ser autodirigido (contra si próprio). O idadismo pode ser implícito ou explícito, dependendo do nível de conscientização sobre nosso próprio viés”^(4)^.

Segundo a Organização Pan-Americana de Saúde (OPAS), o idadismo contra pessoas idosas é mundial, manifestando-se em todas as instituições das sociedades como saúde, assistência social, trabalho e meios de comunicação, nos quais, muitas vezes, há sub-representação. Essa prática compromete o bem-estar, dificulta a implementação de políticas e ações eficazes voltadas à promoção da saúde da população idosa e representa um desafio para o cuidado, que deve priorizar a qualidade de vida, mesmo diante de limitações decorrentes da idade. Tal objetivo torna-se viável à medida que a sociedade reconhece o valor e o potencial das pessoas idosas, superando barreiras resultantes da desvalorização social^(3,4)^.

Em resposta, a OPAS publicou o Relatório Mundial sobre o Idadismo, descrevendo estratégias baseadas em evidências para reduzir o fenômeno e seus impactos^(4)^. Entretanto, estereótipos e preconceitos direcionados às pessoas idosas, frequentemente reproduzidos no cotidiano de profissionais de saúde, influenciam negativamente o cuidado e a resolução de problemas, aumentando a vulnerabilidade dessa população^(5)^.

O idadismo na saúde manifesta-se, entre outros aspectos, na negligência quanto à sexualidade da pessoa idosa, tema para o qual muitos profissionais não se sentem preparados para atuar^(5,6)^. Também se observa o racionamento de cuidados conforme a idade, evidenciado por revisão sistemática que identificou, em 85% de 149 estudos, a idade como determinante para o acesso a procedimentos ou tratamentos médicos específicos^(7)^. Soma-se a isso a escassez de estudos em saúde que incluam a população idosa, apesar da alta prevalência das condições estudadas nesses indivíduos^(4)^. Os impactos do idadismo associam-se à redução da expectativa de vida, piora da saúde física e mental, recuperação lenta de incapacidades, declínio cognitivo e redução da qualidade de vida, com aumento do isolamento e da solidão.

Estudos sobre ensino-aprendizagem evidenciam lacunas na formação em Enfermagem quanto à saúde da pessoa idosa, incluindo déficits na falta de uniformidade em pesquisas sobre idadismo ao longo da graduação: uma revisão sistemática de 2023 identificou resultados inconsistentes sobre o idadismo durante a graduação, em que nove estudos apontaram maior positividade ao longo do curso e sete estudos não apresentaram diferenças significativas, indicando falhas na progressão positiva de percepções ao decorrer dos anos dos graduandos na instituição de ensino^(8)^.

Apesar do reconhecimento da relevância do tema, a produção científica é majoritariamente quantitativa, como mostra revisão de escopo com 46 estudos, dos quais 73,9% eram quantitativos, 10,8% qualitativos e 6,6% mistos^(9)^. Nesse sentido, a análise qualitativa na área da saúde do idoso é de grande valia, especialmente na compreensão dos significados, experiências e comportamentos, aspectos pouco acessíveis pela análise apenas quantitativa^(10)^. Além disso, estudos nacionais indicam que a inclusão da saúde da pessoa idosa na formação em Enfermagem permanece incipiente, restrita a iniciativas pontuais e estratégias pouco consolidadas^(11)^. Evidencia-se, assim, a necessidade de ampliar o debate científico e pedagógico sobre o idadismo na graduação, subsidiando análises mistas e aprofundando discussões sobre a temática.

Vale ressaltar que se utilizou como referencial teórico a teoria das representações sociais de Serge Moscovici, onde é essencial discutir os conceitos: ancoragem e objetivação, processos geradores das representações sociais. Moscovici propõe que a ancoragem consiste em transformar o não-familiar em algo familiar, por meio da classificação e categorização do desconhecido com base no que já é conhecido. Já a objetivação seria transformar o abstrato em algo quase concreto^(12)^.

Para compreender como se entende a sociedade, Moscovici divide o universo em consensual e reificado. O consensual é onde se formam as representações sociais, englobando o senso comum. Já o reificado corresponde ao campo do saber científico^(12)^. A partir disso, a percepção inclina-se para um desses universos, variando conforme o grupo social ao qual se pertence ou no qual está inserido.

A teoria das representações sociais aparece no campo da saúde de forma intensa. Aquele que presta o cuidado é o portador do conhecimento científico, incluindo os enfermeiros, contudo, deve-se considerar que esses portadores do conhecimento científico pertencem a um grupo social e estão sujeitos diariamente a influências vindas dos diferentes meios da sociedade a que pertencem.

Desse modo, o presente estudo visa aportar evidências que contribuam para reduzir a lacuna identificada e teve como objetivo conhecer e analisar as representações sociais de estudantes de graduação em Enfermagem acerca do idadismo e caracterizar o perfil dos estudantes da amostra, visto que eles, futuros enfermeiros, irão atuar na assistência de pessoas idosas e deverão entender e desmistificar o idadismo, tendo a graduação papel vital nesse contexto.

## MÉTODO

### Desenho do Estudo

Para melhor responder aos objetivos, visando compreender as percepções dos estudantes de Enfermagem sobre o idadismo, o presente estudo teve caráter exploratório, descritivo, com metodologia quantitativa e qualitativa dos dados. Foi utilizada a técnica de Análise de Conteúdo fundamentada na Teoria das Representações Sociais de Serge Moscovici como referencial teórico-metodológico^(13)^.

### Local

A pesquisa foi realizada em ambiente virtual, via formulário on-line do Google Forms.

### População e Critérios de Seleção

Participaram alunos de graduação em Enfermagem, ingressantes a partir de 2021, autodeclarados de ambos os sexos, maiores de 18 anos, matriculados em instituições de graduação, públicas e privadas, do estado de São Paulo. Foram excluídos ex-alunos e graduandos menores de idade.

### Instrumento de Coleta de Dados

Foi elaborado um formulário pelo Google Forms com questões sobre o perfil do estudante e questões abertas sobre as percepções frente ao idadismo.

### Procedimento de Coleta de Dados

A coleta de dados foi realizada de Março a Junho de 2024, por meio da técnica de amostragem em cadeia “Bola de Neve”, na qual os participantes foram recrutados a partir dos contatos dos pesquisadores e, posteriormente, solicitados a indicarem novos contatos com as características desejadas. O formulário foi encaminhado via e-mail e/ou Whatsapp, respondido de forma anônima após aceite virtual do Termo de Consentimento Livre e Esclarecido (TCLE), indexado ao início do formulário. O tempo médio de preenchimento do instrumento foi de 7 minutos e o questionário foi respondido em uma única rodada, sem posterior retorno para validação ou complementação dos dados qualitativos.

### Análise e Tratamento dos Dados

Os dados quantitativos foram registrados em planilhas de Excel^®^ e apresentados em tabelas de frequência. As evocações foram processadas no software OpenEvoc para análise qualitativa e, em seguida, examinadas pela análise de conteúdo de Bardin para interpretar os significados atribuídos pelos participantes tendo como base a Teoria das Representações Sociais^(14)^.

### Aspectos Éticos

Atendendo ao Conselho Nacional de Saúde na Resolução n° 466/2012, este projeto foi aprovado pelo Comitê de Ética em Pesquisa da Escola de Enfermagem da USP, Parecer nº 6.691.256 e, atendendo a mesma Resolução, todos os participantes leram e assinaram o TCLE^(15)^. Para garantir a privacidade, todos os participantes foram identificados com a letra G seguida de números sequenciais (G1, G2, G3…).

## RESULTADOS

### Perfil Sociodemográfico dos Estudantes de Enfermagem

O estudo contou com 308 graduandos de Enfermagem, dos quais 242 atenderam aos critérios de inclusão e compuseram a amostra final. Os graduandos tinham entre 18 e 51 anos de idade (média de 23,8 e mediana de 21,5), predominando os declarados do sexo feminino (78,9%; n = 191), de universidade pública (74,0%; n = 179) e do 3° ano de graduação (40,1%; n = 97). Neste grupo, 5,4% (n = 13) dos participantes fizeram outra graduação antes da de enfermagem e 24,4% (n = 59) trabalhavam concomitantemente à graduação. Vale destacar que destes, 65,1% (n = 41) são de universidades privadas. A maioria, 80,6% (n = 195), convivia com pessoas idosas no dia-a-dia e 80,2% (n = 194) conheciam o conceito de idadismo (Tabela 1).

**Tabela 1:** Dados de perfil dos graduandos de enfermagem – São Paulo, SP, Brasil, 2024.

Variáveis	Universidade Pública		Universidade Privada		Total de participantes	
	n	%	n	%	n	%
**Sexo**						
Feminino	138	77,1	53	84,1	191	78,9
Masculino	39	21,8	10	15,9	49	20,3
Prefere não identificar	2	1,1	0	0,0	2	0,8
**Ano de Graduação**						
1 ano	50	28,0	13	20,7	63	26,0
2 ano	31	17,3	9	14,3	40	16,5
3 ano	77	43,0	20	31,7	97	40,1
4 ano	21	11,7	21	33,3	42	17,4
**Outra Graduação**						
Sim	6	3,4	7	11,1	13	5,4
Não	173	96,6	56	88,9	229	94,6
**Trabalho**						
Sim	18	10,1	41	65,1	59	24,4
Não	161	89,9	22	34,9	183	75,6
**Convive com pessoas idosas**						
Sim	140	78,2	55	87,3	195	80,6
Não	39	21,8	8	12,7	47	19,4
**Sabe o significado de Idadismo**						
Sim	153	85,5	41	65,1	194	80,2
Não	26	14,5	22	34,9	48	19,8
Total	179	100,0	63	100,0	242	100,0

Os dados sociodemográficos dos participantes foram apresentados em tabelas de frequência com uma comparação analítica entre unidades públicas e privadas, ao serem avaliados dados significativos na comparação do perfil sociodemográfico entre as instituições.

Ademais, a maioria, 61,2% (n = 148), dos estudantes de Enfermagem considera-se “realista com alguns preconceitos”; em seguida, 25,2% (n = 61) “otimistas e livres de preconceitos”; 8,7% (n = 21) “sem preconceitos”; e, ainda, 2,9% (n = 7) “pessimistas e preconceituosos”. Destaca-se que, entre os graduandos de universidades públicas, a maioria posicionou-se como “Realistas, com alguns preconceitos” (69,3%; n = 124). Já os graduandos das universidades privadas dividiram-se em sua maioria entre “Otimistas e livres de preconceitos”, com 39,7% (n = 25), e “Realistas, com alguns preconceitos”, com 38,1% (n = 24) (Tabela 2).

**Tabela 2:** Percepções dos graduandos de enfermagem sobre pessoas idosas – São Paulo, SP, Brasil 2024.

Variáveis	Universidade Pública		Universidade Privada		Total de participantes	
	n	%	n	%	n	%
Realista, com alguns preconceitos	124	69,3	24	38,1	148	61,2
Otimista e livre de preconceitos	36	20,1	25	39,7	61	25,2
Sem preconceitos	9	5,0	12	19,0	21	8,7
Pessimista e preconceituosa	6	3,4	1	1,6	7	2,9
Outros	4	2,2	1	1,6	5	2,0
Total	179	100,0	63	100,0	242	100,0

### Expressões Sobre o Idadismo Entre Estudantes de Enfermagem

Em busca de identificar a representação social acerca do idadismo, foram solicitadas aos graduandos cinco palavras/expressões que associavam às pessoas idosas. Houve participantes que enviaram mais, outros menos de cinco palavras, sendo obtidas 1.200 citações, que foram inseridas no software openEvoc 1.0.

Apesar de no perfil sociodemográfico e na autodeclaração de percepção dos graduandos haver significativa divergência entre instituições privadas e públicas, nas evocações não havia diferenças relevantes. Nas evocações, 58,7% (n = 37) dos graduandos de instituições privadas apresentaram percepções consideradas positivas acerca da pessoa idosa e do processo de envelhecimento, enquanto que nas instituições públicas foram 63,6% (n = 114). Pela aplicação do teste do qui-quadrado, com p-valor igual a 0,53 > 0,05, não houve diferença estatisticamente significativa entre os tipos de instituições quanto à conotação das respostas.

Do total das representações, as palavras e suas derivações com maior destaque foram: experiência (112), sabedoria (66), cuidado (41), aposentadoria (32), conhecimento (31), fragilidade (29), vivência (24), respeito (22), solidão (22) e família (20). Ao serem indagados sobre quais seriam as palavras mais representativas entre as 5 citadas, as que se destacaram foram: experiência, sabedoria, cuidado, fragilidade e carência.

A partir da leitura e frequência das palavras e expressões descritas pelos entrevistados, foi possível a classificação em 4 categorias: Vivência, Longevidade como processo, Estereótipos e Políticas Públicas, cujas subcategorias e unidades de registro estão apresentadas no Quadro 1.

**Quadro 1:** Expressões dos graduandos de enfermagem sobre as pessoas idosas – São Paulo, SP, Brasil, 2024.

Categoria	Subcategoria	Unidade de registro
1.Vivência 25,1% (n = 301)	a. Experiências de vida (247)	– Experiência – Sabedoria
	b. Compartilhamento de experiências (54)	– Histórias – Conversa
2.Longevidade como processo 30,7% (n = 368)	a. Vulnerabilidade (227)	– Fragilidade – Doenças
	a. Características físicas (100)	– Envelhecimento – Cabelos grisalhos
	b. Estabilidade (41)	– Descanso – Rotina
3.Estereótipos 27,6% (n = 332)	a. Personalidade (192)	– Teimosia – Paciência – Carência
	b. Olhar infantilizado (13)	– Fofos – Velhinho
	c. Evocação ao passado (44)	– Bingo – “Na minha época…”
	d. Laços familiares (83)	– Família – Carinho
4.Políticas públicas 16,6% (n = 199)	a. Promoção (176)	– Cuidado – Aposentadoria
	b. Omissão (26)	– Abandono – Preconceito

1. **Vivência:** Reuniu as evocações relacionadas a sabedoria e conhecimento adquirido pelas pessoas idosas por meio de suas “Experiências de Vida” e a forma como fazem o “Compartilhamento de Experiências” por meio de histórias e conversas, propagando seu legado e suas tradições, com um total de 25,1% (n = 301) de evocações. Depreende-se que esse tópico apresenta relevância por incluir as duas palavras mais recorrentes entre as evocações: “experiência” (112) e “sabedoria” (66). Ao serem indagados sobre o motivo da escolha das palavras evocadas como mais representativas, as percepções foram trazidas conforme os relatos:

*(**Experiência)** é uma palavra especialmente representativa quando se trata da pessoa idosa porque reflete o acúmulo de vivências, aprendizados e conhecimentos ao longo de uma vida. A experiência de vida de uma pessoa idosa é única e pode oferecer perspectivas valiosas sobre diversos aspectos, como família, trabalho, sociedade e superação de desafios. Além disso, a experiência pode ser compartilhada para beneficiar as gerações mais jovens, contribuindo para a preservação de tradições, valores e conhecimentos*. – (G103)

*(**História)** Pois compreende a carga de vida, a forma de se comunicar, a experiência acumulada, a passagem de tradições, a convivência e suas marcas tudo em uma única palavra. Mesmo sendo curta, compreende a beleza e dor que a velhice representa ao meu ver*. – (G162)

2. **Longevidade como processo:** Essa categoria apresenta as repercussões relacionadas ao período de vida em que a pessoa idosa viveu e vive, com a maior porcentagem, de 30,7% (n = 368) de evocações. A categoria foi subdividida em 3 subcategorias: “Vulnerabilidade”, “Características físicas” e “Estabilidade”.

A “Vulnerabilidade” foi a subcategoria mais citada, 61,7% (n = 227), associando o idoso a palavras negativas, referindo-se às incapacidades e limitações, que podem tornar a pessoa idosa dependente e diminuir a qualidade de vida. A subcategoria foi marcada por doenças, dores, limitações físicas, mas também, por fatores psicológicos como a solidão, relacionada a depressão e ansiedade, como nas colocações:

*(**Fragilidade)** Pois na minha opinião os idosos tem maior fragilidade na sua vida, tanto na saúde física, tanto na psicológica, e nas demais necessidades do cotidiano*. – (G42)

*(**Solidão)** Acredito que a maior parte das pessoas de idade avançada, muitas vezes, sentem que suas famílias os colocam como segundo plano e isso os leva a viver em um mundo onde a única companhia possível são a si mesmo*. – (G57)

A subcategoria “Características físicas” esteve relacionada a aparência e condições das pessoas idosas como o cabelo grisalho e o aparecimento de rugas. Além disso, também as nomenclaturas que se relacionam às pessoas idosas em decorrência de suas características, como chamamentos de velho (a) e senhor (a), como segue:

*(**Envelhecimento)** Escolhi essa palavra, pois nos idosos é mais aparente os resultados do processo de envelhecer, como os cabelos grisalhos e a pele mais sensível, ou seja, neles é possível observar as marcas do passar do tempo, o que é bem interessante, pois mostra como de certo modo as mudanças são parte da vida*. – (G56)

Já, a subcategoria “Estabilidade” representa uma visão idealizada do envelhecimento, associando a fase idosa à tranquilidade, descanso e liberdade após longos anos de trabalho. Ademais, a visão de que a quebra desse contexto de calmaria pode gerar descontentamento das pessoas idosas, como é possível observar em:

*(**Rotina)** Geralmente pessoas idosas não gostam de compromissos que atrapalham sua rotina*. – (G166)

*(**Tranquilidade)** A idade traz tranquilidade pela diminuição das responsabilidades e obrigações diárias*. – (G151)

3. **Estereótipos:** Essa categoria apresentou 27,6% (n = 332) de palavras/expressões e uniu as evocações que representavam diferentes estereótipos das pessoas idosas: “Personalidade”, “Olhar infantilizado”, “Evocação ao passado” e “Laços familiares”. As características de personalidade apareceram de forma positiva como paciência e gentileza, mas também negativas como teimosia e carência.

*(**Gentileza)** Porque idosos, mesmo desconhecidos, costumam ser gentis e acolhedores*. – (G218)

*(**Teimosos)** Geralmente idosos não aceitam que estão envelhecendo, logo eles não aceitam ajuda, e não conseguem perceber suas novas limitações tão facilmente*. – (G126)

*(**Infantilização)** As pessoas infantilizam os idosos*. – (G186)

O “olhar infantilizado” dos graduandos em relação às pessoas idosas, reforçado por termos diminutivos para a infantilização da pessoa idosa, como “velhinho” e “vôzinho”. Já a “Evocação ao passado”, com palavras que remetem à velhice - bingo, igreja, almoços na casa dos avós e até mesmo hábitos como evocar o passado para comparar a atualidade - que pode ser observado nas citações:

*(**Igreja)** A população de idosos no Brasil é formada majoritariamente por pessoas com a ideologia cristã. Essa ideologia é muito expressiva no jeito que eles encaram a nova geração e toda a sociedade no geral*. – (G218)

*(**Na minha época…)** Pois denota o nascimento em outra época, os conhecimentos, formas de viver e lidar com o mundo diferentes, denota a passagem do tempo*. – (G158)

E ainda “Laços familiares” descreve a relação dos graduandos com pessoas idosas de sua família, como avós e etc., como pode ser observado na colocação de um graduando:

*(**Afeto)** A palavra afeto representa o carinho que tenho pelos idosos da minha vida e de uma forma geral. Se formos pensar, graças a eles estamos aqui hoje, toda a cultura de uma sociedade, a formação e união das famílias se resultaram das escolhas dos nossos avós e antes deles nossos bisavós e assim sucessivamente. Por isso acredito que a palavra afeto pode representá-los*. - (G55)

4. **Políticas Públicas:** Apresentou um total de 16,6% (n = 199) das palavras/expressões, onde distinguiram-se duas subcategorias, a “Promoção” e a “Omissão”. Na subcategoria “Promoção”, 88,4% (n = 176) das palavras mencionaram políticas públicas voltadas à pessoa idosa, especialmente na saúde e em outras áreas sociais:

*(**Cuidado)** Para mim, o cuidado engloba diversos aspectos, como a atenção especial para doenças crônicas não transmissíveis, que tornam-se mais corriqueiras na velhice, risco aumentados de quedas. A situação de solidão e abandono, que muitos idosos enfrentam, também requer uma maior atenção e mudanças da sociedade*. – (G2)

*(**Respeito)** Porque a pessoa tem mais coisas vividas para dividir, porque já passou por tantas coisas, porque às vezes não é ouvida ou é negligenciada*. – (G119)

Já na subcategoria “Omissão” os participantes associaram a pessoa idosa à vulnerabilidade causada pela falta de políticas públicas, utilizando palavras negativas para denunciar situações adversas enfrentadas pelas pessoas idosas:

*(**Vulnerabilidade Social)** Acredito que na sociedade em que vivemos a vulnerabilidade social é inerente ao idoso, uma vez que os seus direitos são diariamente violados, seja por uma aposentadoria insuficiente, pela falta de acessibilidade nos espaços públicos, entre outros*. – (G65)

*(**Invisibilidade)** Acredito que as pessoas idosas são muitas vezes ignoradas e invisibilizadas pela sociedade. Por exemplo, quando pensamos na infraestrutura urbana precária que dificulta ou até impossibilita a circulação de pessoas idosas. Além disso, existe uma falta de políticas públicas voltadas para esta faixa etária*. – (G78)

## DISCUSSÃO

O grupo dos graduandos que colaboraram com a pesquisa foi composto por maior número de pessoas autodenominadas do sexo feminino, tanto em pesquisas voltadas a estudantes de enfermagem dentro do âmbito do idadismo^(9)^ ou fora dele. Isso demonstra uma feminização da enfermagem, decorrente do histórico da categoria, em que o cuidado assistencial era visto como uma habilidade unicamente feminina.

Foi verificado ainda que a maioria dos participantes de instituições privadas trabalhavam concomitantemente à graduação, enquanto que os de instituições públicas eram uma minoria, o que é compatível com os dados do Sindicato das Mantenedoras de Ensino Superior - SEMESP acerca do perfil dos estudantes do ensino superior no Brasil^(16)^. Esses dados elevados, voltados aos graduandos de instituições privadas, relacionam-se à necessidade de trabalhar para pagar a faculdade, ocasionalmente possibilitados pela menor carga horária da instituição de ensino, o que permite o ensino no período noturno, dando acesso ao trabalho ou, ainda, um estímulo do empregador para o aluno galgar melhores posições no plano de carreira.

Salienta-se que o sexo não apresentou influência significativa na prevalência das palavras evocadas, resultado semelhante ao encontrado em estudo transversal realizado com graduandos de enfermagem sobre idadismo na Jordânia^(15)^. Em contrapartida, outras investigações identificaram diferenças relacionadas ao sexo, evidenciando maior predominância ora entre participantes do sexo feminino, ora entre os do masculino^(8)^.

O tipo de instituição de ensino ou o ano que cursavam a graduação não interferiram na prevalência nas colocações das palavras. Da mesma forma, conviver com pessoas idosas e saber o significado de idadismo, também, não apresentaram diferenças nas citações, mostrando, de certa forma, senso-comum independente de sexo, tipo de instituição e ano de graduação, diferente dos resultados apresentados por Allué-Sierra et al.^(8)^, onde o contato regular ou coabitar com um idoso foram três dos principais determinantes na expressão de atitudes positivas em relação aos idosos.

A experiência de vida das pessoas idosas foi identificada como um conceito relevante nas representações sociais manifestadas, desde a obtenção de sabedoria a partir das vivências, como também a transmissão de aprendizados e tradições advindas do tempo vivido. Essa evocação inicial da pessoa idosa como alguém experiente também foi identificada em outras pesquisas qualitativas acerca do idadismo e com estudantes de graduação de enfermagem, em estudos de âmbito nacional (Ceará) e internacional (Coreia)^(17,18)^.

Outra pesquisa analisou as representações de pessoas idosas acerca de suas vivências e concluiu que é importante que enfermeiros estimem a relevância das experiências de vida das pessoas idosas para o cuidado adequado e humanizado aos pacientes, não enfatizando um cuidado apenas procedimental^(19)^.

Assim, considerar as experiências de pessoas idosas é importante, mas, infelizmente, há um mito de que pessoas longevas são sábias. Essa representação perpetua uma crença de que a experiência acumulada ao longo dos anos traz uma compreensão mais profunda da vida. Embora muitos idosos possuam vasto conhecimento, a sabedoria não é exclusiva da idade avançada, assim como não é obrigatória diante desse avanço^(20)^. Na velhice, a sabedoria pode ser manifestada de diversas maneiras, como a capacidade de refletir sobre o passado com discernimento e a habilidade de lidar com desafios de forma mais ponderada. Ainda, é possível que os graduandos, ao buscarem evitar uma perspectiva idadista, tenham atribuído um valor excessivo à sabedoria em pessoas idosas, refletindo uma tentativa de distanciar-se dos estereótipos consensuais associados ao envelhecimento, caracterizando um possível viés.

Além disto, na sociedade há uma visão do processo de envelhecimento interligado a solidão e dependência; no entanto, também há, muitas vezes, uma veiculação desse pré-conceito pelos próprios serviços de saúde^(21)^. Na pesquisa, houve a representação da longevidade como um processo e, dentro dele, destacou-se a visão da pessoa idosa interligada à vulnerabilidade e fragilidade. A visão de vulnerabilidade da pessoa idosa é senso-comum nessa e em outras pesquisas, como em estudo realizado com questionário autorreferido com graduandos em enfermagem^(22)^; contudo, é importante trazer à memória que muitas pessoas chegam a idades longevas com boa qualidade de vida, independência e autonomia. Dessa forma, o conceito de envelhecimento saudável, em que a pessoa idosa é protagonista de suas ações e escolhas, ainda precisa ser ancorado nos alunos de graduação, bem como o papel do enfermeiro na prevenção e promoção de saúde ao invés de somente tratar as doenças, incapacidades e limitações.

Nesse sentido, percepções estereotipadas sobre as pessoas idosas estão em todos os lugares, seja na mídia, nos atendimentos preferenciais e no setor de saúde. Neste estudo, foram relacionados às pessoas idosas diversos estereótipos, como traços da personalidade positivos (paciência, alegria e gentileza) e negativos (teimosia, carência e conservadorismo). Também foi citado um olhar infantilizado da pessoa idosa e atividades relacionadas com a velhice como bingo, evocar o passado e laços familiares. Diante disso, vale ressaltar que, embora tenham sido identificados estereótipos negativos, os positivos foram os mais frequentes, conforme apontado em uma revisão de escopo. Entre os 46 artigos analisados, 23,5% apresentaram atitudes e percepções positivas, representando a maior proporção dos estudos, seguidos dos negativos (19,6%), mistos (19,6%), neutros (4,3%) e não conclusivos (6,5%)^(9)^.

Estereótipos acerca da velhice ainda são prevalentes e acarretam consequências prejudiciais à sociedade, uma vez que comprometem a autoestima e, consequentemente, a saúde física e mental dos indivíduos. Além disso, resultam na exclusão das pessoas de espaços sociais e laborais, gerando impactos negativos em diversos setores da vida^(23)^. Apesar de no estudo em questão e demais estudos as evocações apresentarem que os graduandos têm um maior contato com pessoas idosas da família^(24,25)^, é preciso ampliar esse olhar para oferecer um cuidado adequado, livre de preconceitos e que promova o envelhecimento saudável.

Ademais, em relação às características físicas, rugas, cabelos brancos e flacidez marcam o fenótipo das pessoas idosas, o qual foi apresentado nas representações deste e demais estudos^(22)^. No entanto, no Brasil, há um certo movimento para envelhecer sem parecer uma pessoa idosa, uma vez que, de acordo com Castro et al.^(26)^, as rugas e cabelos brancos, características evocadas no estudos, são consideradas como um descuido em relação à própria aparência. Dessa forma, as características físicas comuns decorrentes do processo de envelhecimento apresentam-se de forma negativa ao interferir na autoestima da pessoa que está fazendo a transição da fase adulta para a idosa.

Destacou-se a evocação dos alunos sobre políticas públicas voltadas à saúde e à vida em sociedade, muitas vezes negligenciadas, mas fundamentais para o fortalecimento de um envelhecimento saudável. Em outro estudo de representações sociais, essa dimensão relacionada às políticas públicas emergiu após a aplicação de aulas sobre saúde do idoso, não sendo inicialmente identificada entre os participantes^(17)^.

Foram relatados aspectos negativos como a omissão de direitos, que aumentam a vulnerabilidade da pessoa idosa. A OMS define envelhecimento saudável como o processo de desenvolvimento e manutenção da capacidade funcional que permite o bem-estar na idade avançada, enfatizando a importância de políticas que promovam a inclusão social, a acessibilidade aos serviços de saúde e o suporte a estilos de vida que favoreçam a autonomia e a independência dos idosos^(27)^.

Aspectos estes reforçados pelo Estatuto da Pessoa Idosa, em que, de acordo com o Art 1º, “É instituído o Estatuto da Pessoa Idosa, destinado a regular os direitos assegurados às pessoas com idade igual ou superior a 60 (sessenta) anos”, embasando as representações dos participantes acerca de políticas públicas destinadas às pessoas idosas^(28)^.

Considera-se a importância dessas evocações, uma vez que, como futuros enfermeiros, deverão ser, também, um agente político na área da saúde, atuando na defesa da pessoa idosa e na capacitação de mudanças de seus pares, considerando o idoso com suas vulnerabilidades, potencialidades e necessidades, para a melhoria da qualidade de vida das pessoas idosas, em todos os níveis da assistência no território em que praticará sua profissão.

O estudo apresenta como pontos fortes a originalidade e relevância do tema, ainda pouco explorado na formação em Enfermagem, aliado a uma metodologia consistente, combinando abordagens quantitativa e qualitativa fundamentadas na Teoria das Representações Sociais. Conta com uma amostra significativa, o que confere robustez aos achados, organizados em categorias abrangentes que revelam percepções tanto positivas quanto negativas sobre o idadismo. Além disso, destaca-se a inclusão da dimensão de políticas públicas, aspecto inovador na área e as contribuições práticas para estratégias pedagógicas, reforçando a importância da formação crítica e inclusiva dos futuros enfermeiros. Apesar das contribuições, este estudo apresenta limitações que devem ser consideradas, como o uso de uma amostra de conveniência, restrita a estudantes de graduação em Enfermagem, o que limita a extrapolação dos resultados. Ademais, a coleta de dados on-line pode ter favorecido viés de desejabilidade social e, por se tratar de um delineamento transversal, não permite estabelecer causalidade, apenas associações entre as variáveis analisadas. Pesquisas futuras, com amostras maiores e em diversos contextos, poderão aprofundar e ampliar a compreensão sobre o idadismo no âmbito da formação em saúde.

## CONCLUSÃO

De acordo com a análise, foi possível identificar que a maioria dos estudantes de enfermagem tem conhecimento em relação ao conceito de idadismo e aparentam estar abertos para conhecer mais sobre a temática, apresentando percepções otimistas ou realistas sobre o assunto. Embora a palavra “idadismo” não seja diretamente mencionada nas percepções, elas evocam questões atuais da sociedade que permeiam a pessoa idosa e o processo de envelhecimento em diversos âmbitos, como nas políticas públicas e na área da saúde. Além disso, essas percepções também associam a pessoa idosa a sentimentos considerados positivos, como os laços familiares e qualidades como sabedoria, experiência e paciência.

No entanto, diversas palavras negativas ainda se apresentam dentro do imaginário dos participantes acerca da pessoa idosa e diversos estereótipos são evocados como as características físicas e de personalidade negativas, além da correlação da pessoa idosa com o adoecimento, vulnerabilidade e visão infantilizada.

Dessa forma, é de extrema importância que os conhecimentos sobre o idadismo sejam abordados e reforçados aos estudantes de graduação, especialmente os de enfermagem, que poderão estar em contato direto e contínuo com as pessoas idosas, para manter o sentimento positivo em relação a elas, uma vez que é uma população em crescimento mundial e serão certamente alvos de cuidados, quer na área preventiva ou assistencial, em nível ambulatorial, institucional ou domiciliar. Além disso, que os estudantes e futuros enfermeiros possam ser representantes e multiplicadores no combate ao idadismo na sociedade e no futuro profissional.

## Data Availability

Todo o conjunto de dados que dá suporte aos resultados deste estudo foi publicado no próprio artigo.

# Translation: Percepciones sobre la discriminación por edad en estudiantes de licenciatura en enfermería

**DOI:** 10.1590/1980-220X-REEUSP-2024-0411es

**Authors:** Vitória da Cruz Moura, Luciana Mitsue Sakano Niwa, Suely Itsuko Ciosak

**Affiliations:** 1 Universidade de São Paulo, Escola de Enfermagem, São Paulo, SP, Brasil.; 2 Universidade de São Paulo, Escola de Enfermagem, Programa de Pós-Graduação em Enfermagem, São Paulo, SP, Brasil.; 3 Universidade de São Paulo, Escola de Enfermagem, Departamento de Enfermagem em Saúde Coletiva, São Paulo, SP, Brasil.

**Keywords:** Ageísmo, Percepción, Enfermería, Estudiantes de Enfermería

## Abstract

**Objetivo:**

Analizar las representaciones sociales del edadismo entre estudiantes de enfermería de pregrado.

**Método:**

Estudio exploratorio y descriptivo, con enfoque cuantitativo y cualitativo. Los datos se recopilaron a través de Google Forms, investigando el perfil sociodemográfico de los estudiantes de enfermería del estado de São Paulo y sus percepciones sobre el edadismo. Los datos cuantitativos se sometieron a análisis estadístico, mientras que las evocaciones se procesaron mediante software *openEvoca*, basado en la Teoría de las Representaciones Sociales (Moscovici, 2003), y analizado según Bardin (2015).

**Resultados:**

Participaron 242 estudiantes, con una edad promedio de 23,8 años; el 78,9% (n = 191) eran mujeres, el 74,0% (n = 179) asistían a universidades públicas y el 40,1% (n = 97) estaban en su tercer año de estudios de pregrado. Se enumeraron 1200 evocaciones, organizadas en cuatro categorías: “Experiencia”, “La longevidad como proceso”, “Estereotipos” y “Políticas públicas”.

**Conclusión:**

Predominaron las percepciones positivas sobre el edadismo, aunque seguían siendo recurrentes los términos negativos y estereotipados. Estos hallazgos refuerzan la necesidad de estrategias pedagógicas que aborden el edadismo en la formación en enfermería, con el fin de promover actitudes más críticas y positivas hacia las personas mayores.

## INTRODUCCIÓN

El envejecimiento de la población, fenómeno derivado de la transición demográfica caracterizada por la reducción de las tasas de mortalidad y natalidad, ha sido impulsado por el aumento de la esperanza de vida asociado a los avances en los sectores de la salud y la economía^(1)^. Este proceso ha dado lugar a un envejecimiento acelerado en Brasil, donde la proporción de personas de 65 años o más pasó del 9,84% en 2020 a una proyección del 31,0% en 2070, triplicándose en 5 décadas^(2)^. Este escenario representa un logro social, pero plantea retos importantes, sobre todo para los sistemas de salud, que exigen la formulación de políticas públicas integradas que promuevan el envejecimiento saludable. Además, es necesario crear entornos sociales y culturales favorables, capaces de combatir los estereotipos que asocian el envejecimiento con la enfermedad, la dependencia y la inutilidad^(3,4)^.

Este prejuicio relacionado con la edad se denomina etarismo, agismo o edadismo y, en esta investigación, adoptaremos el término etarismo, cuya definición es:

“… se refiere a los estereotipos (la forma en que pensamos), los prejuicios (cómo sentimos) y la discriminación (cómo actuamos), basados en la edad, dirigidos a otros o contra uno mismo. El etarismo puede manifestarse a nivel institucional o interpersonal y puede ser autodirigido (contra uno mismo). El etarismo puede ser implícito o explícito, dependiendo del nivel de conciencia sobre nuestro propio sesgo”^(4)^.

Según la Organización Panamericana de la Salud (OPS), el etarismo contra las personas mayores es mundial y se manifiesta en todas las instituciones de la sociedad, como la salud, la asistencia social, el trabajo y los medios de comunicación, en los que a menudo hay una representación insuficiente. Esta práctica compromete el bienestar, dificulta la implementación de políticas y acciones eficaces orientadas a la promoción de la salud de la población de edad avanzada y representa un desafío para la atención, que debe priorizar la calidad de vida, incluso ante las limitaciones derivadas de la edad. Este objetivo se hace viable a medida que la sociedad reconoce el valor y el potencial de las personas mayores, superando las barreras resultantes de la desvalorización social^(3,4)^.

En respuesta a ello, la OPS publicó el Informe mundial sobre el envejecimiento, en el que se describen estrategias basadas en la evidencia para reducir el fenómeno y sus repercusiones^(4)^. Sin embargo, los estereotipos y prejuicios dirigidos a las personas mayores, que a menudo se reproducen en el día a día de los profesionales de la salud, influyen negativamente en la atención y la resolución de problemas, lo que aumenta la vulnerabilidad de esta población^(5)^.

El etarismo en la salud se manifiesta, entre otros aspectos, en la negligencia con respecto a la sexualidad de las personas mayores, un tema para el que muchos profesionales no se sienten preparados para actuar^(5,6)^. También se observa la racionalización de la atención en función de la edad, como lo demuestra una revisión sistemática que identificó, en el 85% de 149 estudios, la edad como determinante para el acceso a procedimientos o tratamientos médicos específicos^(7)^. A esto se suma la escasez de estudios de salud que incluyan a la población de edad avanzada, a pesar de la alta prevalencia de las afecciones estudiadas en estos individuos^(4)^. Los impactos del etarismo se asocian con la reducción de la esperanza de vida, el empeoramiento de la salud física y mental, la lenta recuperación de las discapacidades, el deterioro cognitivo y la reducción de la calidad de vida, con un aumento del aislamiento y la soledad.

Los estudios sobre enseñanza-aprendizaje evidencian lagunas en la formación en enfermería en lo que respecta a la salud de las personas mayores, incluyendo déficits en la falta de uniformidad en las investigaciones sobre el etarismo a lo largo de la licenciatura: una revisión sistemática de 2023 identificó resultados inconsistentes sobre el etarismo durante la licenciatura, en la que nueve estudios señalaron una mayor positividad a lo largo del curso y siete estudios no presentaron diferencias significativas, lo que indica fallos en la progresión positiva de las percepciones a lo largo de los años de los estudiantes de la institución educativa^(8)^.

A pesar del reconocimiento de la relevancia del tema, la producción científica es mayoritariamente cuantitativa, como muestra una revisión de alcance con 46 estudios, de los cuales el 73,9% eran cuantitativos, el 10,8% cualitativos y el 6,6% mixtos^(9)^. En este sentido, el análisis cualitativo en el ámbito de la salud de las personas mayores es de gran valor, especialmente para comprender los significados, las experiencias y los comportamientos, aspectos poco accesibles mediante el análisis cuantitativo^(10)^. Además, los estudios nacionales indican que la inclusión de la salud de las personas mayores en la formación en enfermería sigue siendo incipiente, restringida a iniciativas puntuales y estrategias poco consolidadas^(11)^. De este modo, se evidencia la necesidad de ampliar el debate científico y pedagógico sobre la discriminación por edad en la licenciatura, subsidiando análisis mixtos y profundizando en las discusiones sobre el tema.

Cabe destacar que se utilizó como referencia teórica la teoría de las representaciones sociales de Serge Moscovici, en la que es esencial discutir los conceptos de anclaje y objetivación, procesos generadores de las representaciones sociales. Moscovici propone que el anclaje consiste en transformar lo desconocido en algo familiar, mediante la clasificación y categorización de lo desconocido basándose en lo que ya se conoce. La objetivación, por su parte, consistiría en transformar lo abstracto en algo casi concreto^(12)^.

Para comprender cómo se entiende la sociedad, Moscovici divide el universo en consensual y reificado. El consensual es donde se forman las representaciones sociales, que engloban el sentido común. El reificado, por su parte, corresponde al campo del conocimiento científico^(12)^. A partir de ahí, la percepción se inclina hacia uno de estos universos, variando según el grupo social al que se pertenece o en el que se está insertado.

La teoría de las representaciones sociales aparece de forma intensa en el campo de la salud. Quien presta la atención es el portador del conocimiento científico, incluidos los enfermeros, sin embargo, hay que tener en cuenta que estos portadores del conocimiento científico pertenecen a un grupo social y están sujetos diariamente a influencias procedentes de los diferentes medios de la sociedad a la que pertenecen.

De este modo, el presente estudio tiene como objetivo aportar evidencias que contribuyan a reducir la brecha identificada y se propuso conocer y analizar las representaciones sociales de los estudiantes de licenciatura en Enfermería sobre el etarismo y caracterizar el perfil de los estudiantes de la muestra, ya que ellos, futuros enfermeros, trabajarán en la asistencia a personas mayores y deberán comprender y desmitificar el etarismo, teniendo la licenciatura un papel vital en este contexto.

## MÉTODO

### Diseño del Estudio

Para responder mejor a los objetivos, con el fin de comprender las percepciones de los estudiantes de Enfermería sobre el etarismo, el presente estudio tuvo un carácter exploratorio y descriptivo, con una metodología cuantitativa y cualitativa de los datos. Se utilizó la técnica de Análisis de Contenido basada en la Teoría de las Representaciones Sociales de Serge Moscovici como referencia teórico-metodológica^(13)^.

### Lugar

La investigación se llevó a cabo en un entorno virtual, mediante un formulario en línea de Google Forms.

### Población y Criterios de Selección

Participaron estudiantes de licenciatura en Enfermería, ingresados a partir de 2021, autodeclarados de ambos sexos, mayores de 18 años, matriculados en instituciones de licenciatura, públicas y privadas, del estado de São Paulo. Se excluyeron exalumnos y estudiantes menores de edad.

### Instrumento de Recolección de Datos

Se elaboró un formulario mediante Google Forms con preguntas sobre el perfil del estudiante y preguntas abiertas sobre las percepciones frente al etarismo.

### Procedimiento de Recolección de Datos

La Recolección de datos se llevó a cabo de marzo a junio de 2024, mediante la técnica de muestreo en cadena “Bola de nieve”, en la que los participantes fueron reclutados a partir de los contactos de los investigadores y, posteriormente, se les pidió que indicaran nuevos contactos con las características deseadas. El formulario se envió por correo electrónico y/o Whatsapp, y se respondió de forma anónima tras la aceptación virtual del Término de Consentimiento Libre y Esclarecido (TCLE), indexado al inicio del formulario. El tiempo medio de cumplimentación del instrumento fue de 7 minutos y el cuestionario se respondió en una sola ronda, sin necesidad de volver a validar o complementar los datos cualitativos.

### Análisis y Tratamiento de Los Datos

Los datos cuantitativos se registraron en hojas de cálculo de Excel® y se presentaron en tablas de frecuencia. Las evocaciones se procesaron en el software OpenEvoc para su análisis cualitativo y, a continuación, se examinaron mediante el análisis de contenido de Bardin para interpretar los significados atribuidos por los participantes basándose en la Teoría de las Representaciones Sociales^(14)^.

### Aspectos Éticos

De conformidad con la Resolución n.º 466/2012 del Consejo Nacional de Salud, este proyecto fue aprobado por el Comité de Ética en Investigación de la Escuela de Enfermería de la USP, Dictamen n.º 6.691.256 y, de conformidad con la misma Resolución, todos los participantes leyeron y firmaron el TCLE^(15)^. Para garantizar la privacidad, todos los participantes fueron identificados con la letra G seguida de números secuenciales (G1, G2, G3…).

## RESULTADOS

### Perfil Sociodemográfico de los Estudiantes de Enfermería

El estudio contó con 308 estudiantes de Enfermería, de los cuales 242 cumplieron los criterios de inclusión y formaron parte de la muestra final. Los estudiantes tenían entre 18 y 51 años de edad (media de 23,8 y mediana de 21,5), predominando los que se declararon mujeres (78,9%; n = 191), de universidades públicas (74,0%; n = 179) y de tercer año de licenciatura (40,1%; n = 97). En este grupo, el 5,4% (n = 13) de los participantes cursaron otra licenciatura antes de la de enfermería y el 24,4% (n = 59) trabajaban al mismo tiempo que cursaban la licenciatura. Cabe destacar que, de estos, el 65,1% (n = 41) procedían de universidades privadas. La mayoría, el 80,6% (n = 195), convivía con personas mayores en su día a día y el 80,2% (n = 194) conocía el concepto de etarismo (Tabla 1).

**Tabla 1:** Datos de perfil de los estudiantes de enfermería, São Paulo – SP, Brasil, 2024.

Variables	Universidad Pública		Universidad Privada		Total de participantes	
	n	%	n	%	n	%
**Sexo**						
Femenino	138	77,1	53	84,1	191	78,9
Masculino	39	21,8	10	15,9	49	20,3
Prefiere no identificar	2	1,1	0	0,0	2	0,8
**Ano de Graduación**						
1 año	50	28,0	13	20,7	63	26,0
2 año	31	17,3	9	14,3	40	16,5
3 año	77	43,0	20	31,7	97	40,1
4 año	21	11,7	21	33,3	42	17,4
**Otra Graduación**						
Si	6	3,4	7	11,1	13	5,4
No	173	96,6	56	88,9	229	94,6
**Trabajo**						
Si	18	10,1	41	65,1	59	24,4
No	161	89,9	22	34,9	183	75,6
**Convive con personas ancianas**						
Si	140	78,2	55	87,3	195	80,6
No	39	21,8	8	12,7	47	19,4
**Sabe el significado de etarismo**						
Si	153	85,5	41	65,1	194	80,2
No	26	14,5	22	34,9	48	19,8
Total	179	100,0	63	100,0	242	100,0

Los datos sociodemográficos de los participantes se presentaron en tablas de frecuencia con una comparación analítica entre unidades públicas y privadas, al evaluarse datos significativos en la comparación del perfil sociodemográfico entre las instituciones.

Además, la mayoría, el 61,2% (n = 148), de los estudiantes de Enfermería se considera “realista con algunos prejuicios”; seguidos por un 25,2% (n = 61) que se consideran “optimistas y libres de prejuicios”; un 8,7% (n = 21) “sin prejuicios”; y un 2,9% (n = 7) “pesimistas y prejuiciosos”. Cabe destacar que, entre los estudiantes de universidades públicas, la mayoría se posicionó como “realistas, con algunos prejuicios” (69.3%; n = 124). Por su parte, los estudiantes de universidades privadas se dividieron en su mayoría entre “optimistas y sin prejuicios”, con un 39,7% (n = 25), y “realistas, con algunos prejuicios”, con un 38,1% (n = 24) (Tabla 2).

**Tabla 2:** Percepciones de los estudiantes de enfermería sobre las personas mayores – São Paulo, SP, Brasil, 2024.

Variables	Universidad Pública		Universidad Privada		Total de participantes	
	n	%	n	%	n	%
Realista, con algunos prejuicios	124	69,3	24	38,1	148	61,2
Optimista y libre de prejuicios	36	20,1	25	39,7	61	25,2
Sin prejuicios	9	5,0	12	19,0	21	8,7
Pesimista y prejuiciada	6	3,4	1	1,6	7	2,9
Otros	4	2,2	1	1,6	5	2,0
Total	179	100,0	63	100,0	242	100,0

### Expresiones Sobre el Etarismo Entre Estudiantes de Enfermería

Con el fin de identificar la representación social sobre el etarismo, se pidió a los estudiantes de licenciatura que proporcionaran cinco palabras/expresiones que asociaran con las personas mayores. Algunos participantes enviaron más, otros menos de cinco palabras, obteniéndose 1200 citas, que se introdujeron en el software openEvoc 1.0.

Aunque en el perfil sociodemográfico y en la autodeclaración de percepción de los estudiantes de último año había una divergencia significativa entre las instituciones privadas y públicas, en las evocaciones no hubo diferencias relevantes. En las evocaciones, el 58.7% (n = 37) de los estudiantes de instituciones privadas presentaron percepciones consideradas positivas sobre las personas mayores y el proceso de envejecimiento, mientras que en las instituciones públicas fueron el 63.6% (n = 114). Mediante la aplicación de la prueba de Chi-cuadrado, con un valor p igual a 0,53 > 0,05, no hubo diferencias estadísticamente significativas entre los tipos de instituciones en cuanto a la connotación de las respuestas.

Del total de representaciones, las palabras y sus derivaciones más destacadas fueron: experiencia (112), sabiduría (66), cuidado (41), jubilación (32), conocimiento (31), fragilidad (29), vivencia (24), respeto (22), soledad (22) y familia (20). Cuando se les preguntó cuáles serían las palabras más representativas entre las cinco citadas, las que más destacaron fueron: experiencia, sabiduría, cuidado, fragilidad y carencia.

A partir de la lectura y la frecuencia de las palabras y expresiones descritas por los entrevistados, fue posible clasificarlas en cuatro categorías: Vivencia, Longevidad como proceso, Estereotipos y Políticas públicas, cuyas subcategorías y unidades de registro se presentan en el Cuadro 1:

**Cuadro 1:** Expresiones de los estudiantes de enfermería sobre las personas mayores – São Paulo, SP, Brasil, 2024.

Categoría	Subcategoría	Unidad de registro
1.Vivencia 25,1% (n = 301)	a. Experiencias de vida (247)	– Experiencia – Sabiduría
	b. Compartir de experiencias (54)	– Historias – Conversaciones
2.Longevidade como proceso 30,7% (n = 368)	a. Vulnerabilidad (227)	– Fragilidad – Enfermedad
	a. Características físicas (100)	– Envejecimiento – Cabellos grises
	b. Estabilidad (41)	– Descanso – Rutina
3.Estereotipos 27,6% (n = 332)	a. Personalidad (192)	– Terquedad – Paciencia – Carencia
	b. Mirada infantilizada (13)	– Tiernos – Viejitos
	c. Evocación del pasado (44)	– Bingo – “En mi época…”
	d. Lazos familiares (83)	– Familia – Cariño
4.Políticas públicas 16,6% (n = 199)	a. Promoción (176)	– Cuidado – Jubilación
	b. Omisión (26)	– Abandono – Prejuicio

1. V**ivencia**: Reunió las evocaciones relacionadas con la sabiduría y el conocimiento adquiridos por las personas mayores a través de sus “experiencias de vida” y la forma en que “comparten experiencias” a través de historias y conversaciones, propagando su legado y sus tradiciones, con un total del 25,1% (n = 301) de evocaciones. Se deduce que este tema es relevante porque incluye las dos palabras más recurrentes entre las evocaciones: “experiencia” (112) y “sabiduría” (66). Cuando se les preguntó sobre el motivo de la elección de las palabras evocadas como más representativas, las percepciones se reflejaron en los siguientes relatos:

*(**Vivencia**) es una palabra especialmente representativa cuando se trata de las personas mayores porque refleja la acumulación de vivencias, aprendizajes y conocimientos a lo largo de una vida. La experiencia de vida de una persona mayor es* única *y puede ofrecer perspectivas valiosas sobre diversos aspectos, como la familia, el trabajo, la sociedad y la superación de retos. Además, la experiencia puede compartirse para beneficiar a las generaciones más jóvenes, contribuyendo a la preservación de tradiciones, valores y conocimientos*. – (G103)

*(**Historia**) Porque comprende la carga de la vida, la forma de comunicarse, la experiencia acumulada, la transmisión de tradiciones, la convivencia y sus huellas, todo en una sola palabra. Aunque es breve, comprende la belleza y el dolor que, en mi opinión, representa la vejez*. – (G162)

2. **La longevidad como proceso:** Esta categoría presenta las repercusiones relacionadas con el período de vida que la persona mayor ha vivido y vive, con el mayor porcentaje, 30,7% (n = 368) de evocaciones. La categoría se subdividió en tres subcategorías: “Vulnerabilidad”, “Características físicas” y “Estabilidad”.

“Vulnerabilidad” fue la subcategoría más citada, con un 61,7% (n = 227), asociando a las personas mayores con palabras negativas, en referencia a las incapacidades y limitaciones que pueden hacer que las personas mayores sean dependientes y disminuir su calidad de vida. La subcategoría se caracterizó por enfermedades, dolores, limitaciones físicas, pero también por factores psicológicos como la soledad, relacionada con la depresión y la ansiedad, como en las siguientes afirmaciones:

*(**Fragilidad**) Porque, en mi opinión, las personas mayores tienen una mayor fragilidad en su vida, tanto en lo que se refiere a la salud física como a la psicológica, y en el resto de necesidades cotidianas*. – (G42)

*(**Soledad**) Creo que la mayoría de las personas de edad avanzada, a menudo, sienten que sus familias las dejan en un segundo plano y eso las lleva a vivir en un mundo donde la* única *compañía posible es ellas mismas*. – (G57)

La subcategoría “Características físicas” se relacionó con la apariencia y las condiciones de las personas mayores, como el cabello gris y la aparición de arrugas. Además, también se incluyeron las denominaciones relacionadas con las personas mayores debido a sus características, como los términos “viejo” y “señor”, tal y como se muestra a continuación:

*(**Envejecimiento**) Elegí esta palabra porque en las personas mayores son más evidentes los resultados del proceso de envejecimiento, como el cabello canoso y la piel más sensible, es decir, en ellas se pueden observar las marcas del paso del tiempo, lo cual es muy interesante, ya que muestra cómo, en cierto modo, los cambios son parte de la vida*. – (G56)

Por su parte, la subcategoría “Estabilidad” representa una visión idealizada del envejecimiento, asociando la vejez con la tranquilidad, el descanso y la libertad tras largos años de trabajo. Además, la visión de que la ruptura de este contexto de calma puede generar descontento en las personas mayores, como se puede observar en:

*(**Rutina**) Por lo general, a las personas mayores no les gustan los compromisos que perturban su rutina*. – (G166)

*(**Tranquilidad**) La edad trae tranquilidad por la disminución de las responsabilidades y obligaciones diarias*. – (G151)

3. **Estereotipos**: Esta categoría presentó el 27,6% (n = 332) de palabras/expresiones y reunió las evocaciones que representaban diferentes estereotipos de las personas mayores: “Personalidad”, “Mirada infantilizada”, “Evocación del pasado” y “Lazos familiares”. Las características de la personalidad aparecieron de forma positiva, como la paciencia y la amabilidad, pero también negativa, como la terquedad y la dependencia.

*(**Amabilidad**) Porque las personas mayores, incluso las desconocidas, suelen ser amables y acogedoras*. – (G218)

*(**Tercos**) Por lo general, las personas mayores no aceptan que están envejeciendo, por lo que no aceptan ayuda y no logran percibir sus nuevas limitaciones tan fácilmente*. – (G126)

*(**Infantilización**) La gente infantiliza a las personas mayores*. – (G186)

La “mirada infantilizada” de los graduados hacia las personas mayores, reforzada por términos diminutivos para infantilizar a las personas mayores, como “viejito” y “abuelito”. Por otro lado, la “evocación del pasado”, con palabras que remiten a la vejez — bingo, iglesia, almuerzos en casa de los abuelos e incluso hábitos como evocar el pasado para compararlo con el presente—, que puede observarse en las citas:

*(**Iglesia**) La población de personas mayores en Brasil está formada en su mayoría por personas con ideología cristiana. Esta ideología es muy significativa en la forma en que ven a la nueva generación y a toda la sociedad en general*. – (G218)

*(**En mi*** época…*) Porque denota el nacimiento en otra* época, *los conocimientos, las formas de vivir y de lidiar con el mundo diferentes, denota el paso del tiempo*. – (G158)

Y además, “Lazos familiares” describe la relación de los graduados con las personas mayores de su familia, como los abuelos, etc., como se puede observar en la afirmación de un graduado:

*(**Afecto**) La palabra afecto representa el cariño que siento por las personas mayores de mi vida y en general. Si lo pensamos bien, gracias a ellos estamos aquí hoy, toda la cultura de una sociedad, la formación y la unión de las familias son el resultado de las decisiones de nuestros abuelos y, antes de ellos, de nuestros bisabuelos, y así sucesivamente. Por eso creo que la palabra afecto puede representarlos*. – (G55)

4. **Políticas públicas**: Representó un total del 16,6% (n = 199) de las palabras/expresiones, donde se distinguieron dos subcategorías, “Promoción” y “Omisión”. En la subcategoría “Promoción”, el 88.4% (n = 176) de las palabras mencionaban políticas públicas dirigidas a las personas mayores, especialmente en salud y otras áreas sociales:

*(**Cuidado**) Para mí, el cuidado abarca varios aspectos, como la atención especial a las enfermedades crónicas no transmisibles, que se vuelven más comunes en la vejez, y el aumento del riesgo de caídas. La situación de soledad y abandono a la que se enfrentan muchas personas mayores también requiere una mayor atención y cambios en la sociedad*. – (G2)

*(**Respeto**) Porque la persona tiene más experiencias que compartir, porque ha pasado por muchas cosas, porque a veces no se le escucha o se le descuida*. – (G119)

En la subcategoría “Omisión”, los participantes asociaron a las personas mayores con la vulnerabilidad causada por la falta de políticas públicas, utilizando palabras negativas para denunciar las situaciones adversas a las que se enfrentan las personas mayores:

*(**Vulnerabilidad social**) Creo que en la sociedad en la que vivimos la vulnerabilidad social es inherente a las personas mayores, ya que sus derechos se violan a diario, ya sea por una jubilación insuficiente, por la falta de accesibilidad en los espacios públicos, entre otros*. – (G65)

*(**Invisibilidad**) Creo que las personas mayores son a menudo ignoradas e invisibilizadas por la sociedad. Por ejemplo, cuando pensamos en la precaria infraestructura urbana que dificulta o incluso imposibilita la circulación de las personas mayores. Además, existe una falta de políticas públicas dirigidas a este grupo de edad*. – (G78)

## DISCUSIÓN

El grupo de estudiantes de licenciatura que colaboró con la investigación estaba compuesto por un mayor número de personas que se autodenominaban mujeres, tanto en investigaciones dirigidas a estudiantes de enfermería dentro del ámbito del etarismo^(9)^ como fuera de él. Esto demuestra una feminización de la enfermería, derivada de la historia de la categoría, en la que el cuidado asistencial se consideraba una habilidad exclusivamente femenina.

También se observó que la mayoría de los participantes de instituciones privadas trabajaban al mismo tiempo que cursaban la licenciatura, mientras que los de instituciones públicas eran una minoría, lo que es compatible con los datos del Sindicato de Mantenedores de Educación Superior (SEMESP) sobre el perfil de los estudiantes de educación superior en Brasil^(16)^. Estos datos elevados, referidos a los estudiantes de instituciones privadas, se relacionan con la necesidad de trabajar para pagar la universidad, lo que en ocasiones es posible gracias a la menor carga horaria de la institución educativa, que permite la enseñanza en el período nocturno, dando acceso al trabajo o, incluso, un estímulo del empleador para que el estudiante alcance mejores posiciones en su carrera profesional.

Cabe destacar que el sexo no influyó de manera significativa en la prevalencia de las palabras evocadas, un resultado similar al encontrado en un estudio transversal realizado con estudiantes de enfermería sobre el etarismo en Jordania^(15)^.Por el contrario, otras investigaciones identificaron diferencias relacionadas con el sexo, evidenciando una mayor predominancia ora entre las participantes del sexo femenino, ora entre las del masculino^(8)^.

El tipo de institución educativa o el año en que cursaban la licenciatura no interfirieron en la prevalencia de las palabras evocadas. Del mismo modo, convivir con personas mayores y conocer el significado del etarismo tampoco presentaron diferencias en las citas, lo que muestra, en cierta medida, un sentido común independiente del sexo, el tipo de institución y el año de licenciatura, a diferencia de los resultados presentados por Allué-Sierra et al.^(8)^, donde el contacto regular o la convivencia con una persona mayor fueron tres de los principales determinantes en la expresión de actitudes positivas hacia las personas mayores.

Las vivencias de las personas mayores se identificaron como un concepto relevante en las representaciones sociales manifestadas, desde la obtención de sabiduría a partir de las vivencias, hasta la transmisión de aprendizajes y tradiciones derivadas del tiempo vivido. Esta evocación inicial de la persona mayor como alguien con experiencia también se identificó en otras investigaciones cualitativas sobre el etarismo y con estudiantes de licenciatura en enfermería, en estudios de ámbito nacional (Ceará) e internacional (Corea)^(17,18)^.

Otra investigación analizó las representaciones de las personas mayores sobre sus experiencias y concluyó que es importante que los enfermeros valoren la relevancia de las experiencias de vida de las personas mayores para el cuidado adecuado y humanizado de los pacientes, sin enfatizar únicamente el cuidado procedimental^(19)^.

Por lo tanto, es importante tener en cuenta las experiencias de las personas mayores, pero, lamentablemente, existe el mito de que las personas longevas son sabias. Esta representación perpetúa la creencia de que la experiencia acumulada a lo largo de los años aporta una comprensión más profunda de la vida. Aunque muchas personas mayores poseen un vasto conocimiento, la sabiduría no es exclusiva de la edad avanzada, así como tampoco es obligatoria ante este avance^(20)^. En la vejez, la sabiduría puede manifestarse de diversas maneras, como la capacidad de reflexionar sobre el pasado con discernimiento y la habilidad de lidiar con los desafíos de una manera más ponderada. Además, es posible que los estudiantes, al tratar de evitar una perspectiva discriminatoria hacia la edad, hayan atribuido un valor excesivo a la sabiduría de las personas mayores, lo que refleja un intento de distanciarse de los estereotipos consensuados asociados al envejecimiento, lo que caracteriza un posible sesgo.

Además, en la sociedad existe una visión del proceso de envejecimiento vinculada a la soledad y la dependencia; sin embargo, a menudo también se transmite este prejuicio por parte de los propios servicios de salud^(21)^. En la investigación, se representó la longevidad como un proceso y, dentro de él, se destacó la visión de la persona mayor vinculada a la vulnerabilidad y la fragilidad. La visión de vulnerabilidad de la persona mayor es un lugar común en esta y otras investigaciones, como en un estudio realizado con un cuestionario autorreferido con estudiantes de licenciatura en enfermería^(22)^; sin embargo, es importante recordar que muchas personas llegan a edades avanzadas con buena calidad de vida, independencia y autonomía. De este modo, el concepto de envejecimiento saludable, en el que la persona mayor es protagonista de sus acciones y elecciones, aún debe ser afianzado en los estudiantes de licenciatura, así como el papel del enfermero en la prevención y promoción de la salud, en lugar de solo tratar las enfermedades, discapacidades y limitaciones.

En este sentido, las percepciones estereotipadas sobre las personas mayores están en todas partes, ya sea en los medios de comunicación, en la atención preferencial y en el sector de la salud. En este estudio, se relacionaron con las personas mayores varios estereotipos, como rasgos de personalidad positivos (paciencia, alegría y amabilidad) y negativos (terquedad, carencia y conservadurismo). También se mencionó una visión infantilizada de las personas mayores y actividades relacionadas con la vejez, como el bingo, evocar el pasado y los lazos familiares. Ante esto, cabe destacar que, aunque se identificaron estereotipos negativos, los positivos fueron los más frecuentes, según se señala en una revisión de alcance. Entre los 46 artículos analizados, el 23,5% presentaba actitudes y percepciones positivas, lo que representa la mayor proporción de los estudios, seguidos de los negativos (19,6%), mixtos (19,6%), neutros (4,3%) y no concluyentes (6,5%)^(9)^.

Los estereotipos sobre la vejez siguen siendo frecuentes y tienen consecuencias perjudiciales para la sociedad, ya que comprometen la autoestima y, en consecuencia, la salud física y mental de las personas. Además, dan lugar a la exclusión de las personas de los espacios sociales y laborales, lo que genera impactos negativos en diversos ámbitos de la vida^(23)^. Aunque en el estudio en cuestión y en otros estudios las evocaciones muestran que los estudiantes universitarios tienen un mayor contacto con las personas mayores de la familia^(24,25)^, es necesario ampliar esta perspectiva para ofrecer una atención adecuada, libre de prejuicios y que promueva un envejecimiento saludable.

Además, en relación con las características físicas, las arrugas, las canas y la flacidez marcan el fenotipo de las personas mayores, que se presentó en las representaciones de este y otros estudios^(22)^. Sin embargo, en Brasil existe una cierta tendencia a envejecer sin parecer una persona mayor, ya que, según Castro et al.^(26)^, las arrugas y las canas, características evocadas en los estudios, se consideran un descuido en relación con la propia apariencia. De este modo, las características físicas comunes derivadas del proceso de envejecimiento se presentan de forma negativa al interferir en la autoestima de la persona que está haciendo la transición de la edad adulta a la vejez.

Se destacó la evocación de los alumnos sobre las políticas públicas orientadas a la salud y a la vida en sociedad, a menudo descuidadas, pero fundamentales para el fortalecimiento de un envejecimiento saludable. En otro estudio de representaciones sociales, esta dimensión relacionada con las políticas públicas surgió tras la impartición de clases sobre la salud de las personas mayores, no habiéndose identificado inicialmente entre los participantes^(17)^.

Se señalaron aspectos negativos como la omisión de derechos, que aumentan la vulnerabilidad de las personas mayores. La OMS define el envejecimiento saludable como el proceso de desarrollo y mantenimiento de la capacidad funcional que permite el bienestar en la vejez, enfatizando la importancia de políticas que promuevan la inclusión social, la accesibilidad a los servicios de salud y el apoyo a estilos de vida que favorezcan la autonomía y la independencia de las personas mayores^(27)^.

Estos aspectos se refuerzan en el Estatuto de la Persona Mayor, en el que, de acuerdo con el artículo 1, “Se instituye el Estatuto de la Persona Mayor, destinado a regular los derechos garantizados a las personas de 60 (sesenta) años o más”, lo que respalda las representaciones de los participantes sobre las políticas públicas destinadas a las personas mayores^(28)^.

Se considera la importancia de estas evocaciones, ya que, como futuros enfermeros, también deberán ser agentes políticos en el ámbito de la salud, actuando en defensa de las personas mayores y capacitando a sus pares para el cambio, considerando a las personas mayores con sus vulnerabilidades, potencialidades y necesidades, para mejorar la calidad de vida de las personas mayores, en todos los niveles de la asistencia en el territorio en el que ejercerán su profesión.

El estudio presenta como puntos fuertes la originalidad y relevancia del tema, aún poco explorado en la formación en Enfermería, junto con una metodología consistente, que combina enfoques cuantitativos y cualitativos basados en la Teoría de las Representaciones Sociales. Cuenta con una muestra significativa, lo que confiere solidez a los hallazgos, organizados en categorías amplias que revelan percepciones tanto positivas como negativas sobre el etarismo. Además, destaca la inclusión de la dimensión de las políticas públicas, un aspecto innovador en el área, y las contribuciones prácticas a las estrategias pedagógicas, lo que refuerza la importancia de la formación crítica e inclusiva de los futuros enfermeros. A pesar de sus contribuciones, este estudio presenta limitaciones que deben tenerse en cuenta, como el uso de una muestra de conveniencia, restringida a estudiantes de licenciatura en Enfermería, lo que limita la extrapolación de los resultados. Además, la recopilación de datos en línea puede haber favorecido el sesgo de deseabilidad social y, al tratarse de un diseño transversal, no permite establecer causalidad, solo asociaciones entre las variables analizadas. Investigaciones futuras, con muestras más grandes y en diversos contextos, podrán profundizar y ampliar la comprensión sobre el etarismo en el ámbito de la formación en salud.

## CONCLUSIÓN

Según este análisis, se pudo identificar que la mayoría de los estudiantes de enfermería tienen conocimientos sobre el concepto de etarismo y parecen estar abiertos a aprender más sobre el tema, presentando percepciones optimistas o realistas al respecto. Aunque la palabra “etarismo” no se menciona directamente en las percepciones, estas evocan cuestiones actuales de la sociedad que afectan a las personas mayores y al proceso de envejecimiento en diversos ámbitos, como las políticas públicas y el área de la salud. Además, estas percepciones también asocian a las personas mayores con sentimientos considerados positivos, como los lazos familiares y cualidades como la sabiduría, la experiencia y la paciencia.

Sin embargo, aún existen varias palabras negativas en el imaginario de los participantes sobre las personas mayores y se evocan diversos estereotipos, como características físicas y de personalidad negativas, además de la correlación de las personas mayores con la enfermedad, la vulnerabilidad y una visión infantilizada.

Por lo tanto, es de suma importancia que los conocimientos sobre el etarismo se aborden y refuercen entre los estudiantes de licenciatura, especialmente los de enfermería, que pueden estar en contacto directo y continuo con las personas mayores, para mantener un sentimiento positivo hacia ellas, ya que se trata de una población en crecimiento a nivel mundial y que sin duda serán objeto de cuidados, ya sea en el ámbito preventivo o asistencial, a nivel ambulatorio, institucional o domiciliario. Además, que los estudiantes y futuros enfermeros puedan ser representantes y multiplicadores en la lucha contra el etarismo en la sociedad y en su futuro profesional.

## Data Availability

Todo el conjunto de datos que apoya los resultados de este estudio fue publicado en el propio artículo.
